# Supplementary material for: Proteomic investigation of neural stem cell to oligodendrocyte precursor cell differentiation reveals phosphorylation-dependent Dclk1 processing
Source: Cell Mol Life Sci. 2023 Aug 18;80(9):260. doi: 10.1007/s00018-023-04892-8 (PMC10439241; doi:10.1007/s00018-023-04892-8)
Supplement: Supplementary file 1 — Supplementary file1 Supplementary Figures and uncropped western blots and agarose gels (PDF 26074 KB) [file 18_2023_4892_MOESM1_ESM.pdf]

Supplementary Information

Proteomic investigation of neural stem cell to oligodendrocyte precursor cell differentiation reveals phosphorylation-dependent Dclk1 processing

Robert Hardt, Alireza Dehghani, Carmen Schoor, Markus Gödderz, Nur Cengiz Winter, Shiva Ahmadi, Ramesh Sharma, Karin Schork, Martin Eisenacher, Volkmar Gieselmann, Dominic Winter

Supplementary Figure Legends

Figure S1: Immunofluorescence analysis of dissociated spheres during neurosphere to oligosphere differentiation.
Samples were taken at days 0, 3, 6, and 9, spheres dissociated and grown in adherent cultures on cover slips. Cover slips were stained with the respective antibodies and analyzed for the presence of astrocytes (glial fibrillary acidic protein, Gfap), neurons (β3-tubulin), microglia (cell surface glycoprotein F4/80), and oligodendrocytes (myelin basic protein, Mbp) by fluorescence microscopy.

Figure S2: Post-processing of TMT data from neurosphere to oligosphere differentiation.
A) Abundances (log2 values) of individual reporter ion channels of TMT data before normalization. **B)** Abundances (log2 values) of individual reporter ion channels of TMT data after LOESS and subsequent batch normalization. Batch normalization was based on a linear regression model to reduce batch effects between replicates. **C)** Pearson correlation heatmap for individual replicates and samples. **D)** Row-wise hierarchical clustering heatmap (based on Spearman correlation distance and complete linkage) for individual replicates and samples. Shown are z-scored (row) normalized log2 abundances.

Figure S3: Results of paired t-tests between day 0 and individual subsequent time points.
Shown are individual volcano plots for each comparison, -log10 p-values and log2 fold-change ratios are shown. Vertical lines indicate a fold-change of > 2 or < 0.5, horizontal lines an adjusted p-value of 0.05 (back-transformed to the scale of the original p-values). Numbers of significantly up- or down-regulated proteins are indicated (cutoff: adjusted p-value < 0.05 and fold change > 2 or < 0.5). **A)** Day 3 vs. day 0. **B)** Day 6 vs. day 0. **C)** Day 9 vs. day 0. **D)** Day 12 vs. day 0. **E)** Day 15 vs. day 0.

Figure S4: Gene Ontology (GO) analyses for individual clusters of the TMT dataset and investigation of PurA/alpha-Tubulin-antibody cross-reactivity.
A-D) For all individual clusters (Fig. 2B), GO analyses were performed. Fold-enrichment and FDR p-values of all results for the GO categories "Biological Process" (left) and "Cellular Component" (right) are shown. Only categories with an FDR p-value < 0.01 and a fold enrichment > 4 are indicated. Data are only shown for clusters 1-4, as analysis of clusters 5 to 7 resulted in few or no significantly enriched GO terms (for all results, see Table S1). **E)** Western blot analysis of PurA and alpha-Tubulin expression in untransfected and PurA-transfected HeLa cells. Increase in PurA-signal intensity confirms lack of cross-reactivity of PurA and alpha-Tubulin antibodies.

Figure S5: Quality control plots for the dimethyl labeling based phosphoproteomic analysis of neurosphere to oligosphere differentiation.
A/B) Density plots showing the distribution of normalized log2 ratios of quantified protein groups **(A)** and class I phosphosites **(B)** for individual replicates of the different time point comparisons. **C/D)** Boxplots showing the distribution of normalized log2 ratios of quantified protein groups **(C)** and class I phosphosites **(D)** for individual replicates of the different time point comparisons.

Figure S6: Sequence pattern analysis of regulated phosphorylation sites.
IceLogo[38] plots for up- and downregulated class I phosphosites in comparisons of different time points.

Figure S7: Expression analysis of different Dclk1 isoforms.
A) Schematic representation of the four main Dclk1-isoforms, Dclk1 long (Dclk1-L), Dclk1 short (Dclk1-S), Doublecortin-like (Dcl), and CaMK Related Peptide (Carp). Functional domains are highlighted in color. DCX = Doublecortin domain, SP = SP-rich domain, Kinase = Kinase-domain. **B)** TMT signal intensities of 5 unique Dclk1-peptides during neurosphere to oligosphere differentiation. Shown are mean values (n = 7) of (median-) normalized peptide intensities scaled to a 0 to 1 interval. **C/D)** Western blot analysis of Dclk1-isoform expression in different cell lines.

Figure S8: Proteomic analysis of Dclk1 isoform interactomes.
A) Principal component analysis (PCA) of individual samples. **B)** Clustered heatmap analysis of individual samples (Manhattan distance, complete linkage clustering). Analyses shown in (A/B) are based on normalized log2 intensities after missing value imputation.

Figure S9: Differential expression analysis for individual Dclk1 isoform interactomes and overlap determination for proteins which are differentially interacting with individual Dclk1 isoforms.
Binary comparisons for protein abundances of individual interactomes for different BirA*-Dclk1 constructs. Significantly differentially abundant proteins were determined based on a fold-change (log2 $\geq |0.58|$) and adjusted p-value (q-value) cutoff (< 0.05). **A)** SK vs. K; **B)** DSK vs. DS; **C)** DSK vs. SK; **D)** DSK vs. K; **E)** DS vs. SK; **F)** DS vs. K. D: Dcx domain; K: kinase domain; S: SP-rich domain. Colored dots indicate regulated proteins (up = red, down = blue), numbers in the same color represent the number of proteins belonging to the respective category. **G/H)** Comparison of all proteins which were significantly upregulated for constructs containing a Dcx domain, therefore localizing to microtubules **(G)**, or such without this domain, localizing to the cytosol or the nucleus **(H)**. Venn diagrams were generated with the R package VennDiagram (version 1.7.3).

Figure S10: Analysis of Dclk1 isoform interactome phosphosites.
A) Principal component analysis (PCA) of individual samples. **B)** Clustered heatmap of individual samples (Manhattan distance, complete linkage clustering). Analyses shown in (A/B) are based on normalized log2 intensities after missing value imputation. **C)** Count of regulated class I phosphosites per construct. Shown are mean values +/- SD (n = 3).

Figure S11: Differential expression analysis of phosphorylation sites identified in individual Dclk1 isoform interactomes.
Binary abundance comparisons for individual phosphosites identified in the interactomes of different BirA*-Dclk1 constructs. Significantly regulated phosphosites were determined based on a fold-change (log2 $\geq |0.58|$) and adjusted p-value (q-value) cutoff (< 0.05). Only fusion proteins containing the Dclk1 kinase domain were considered. **A)** DSK vs. K; **B)** DSK vs. SK; **C)** SK vs. K. D: Dcx domain; K: kinase domain; S: SP-rich domain. Colored dots indicate regulated proteins (up = red, down = blue), numbers in the same color represent the number of proteins belonging to the respective category. **D/E/F)** Comparison of significantly regulated phosphosites with the

neurosphere to oligosphere differentiation dimethyl dataset. All proteins regulated in the BioID dataset are compared to the proteins regulated in the dimethyl dataset at day 7 vs. 0 and day 14 vs 0, respectively (D). Proteins which are upregulated during differentiation are matched to interaction partners of microtubule-localized Dclk1 (E), while such being downregulated are matched to cytosolic/nuclear (F). Venn diagrams were generated with the R package VennDiagram (version 1.7.3)

Figure S12: Identification of potential Dclk1 cleavage sites in the SP-rich region.
Retention time distributions of semi-tryptic Dclk1 peptides found in the SP-rich region. Peptides are sorted by amino acid start position in ascending order. **A)** Peptides identified between amino acid 305-326. **B)** Peptides identified between amino acid 346-360.

Figure S1

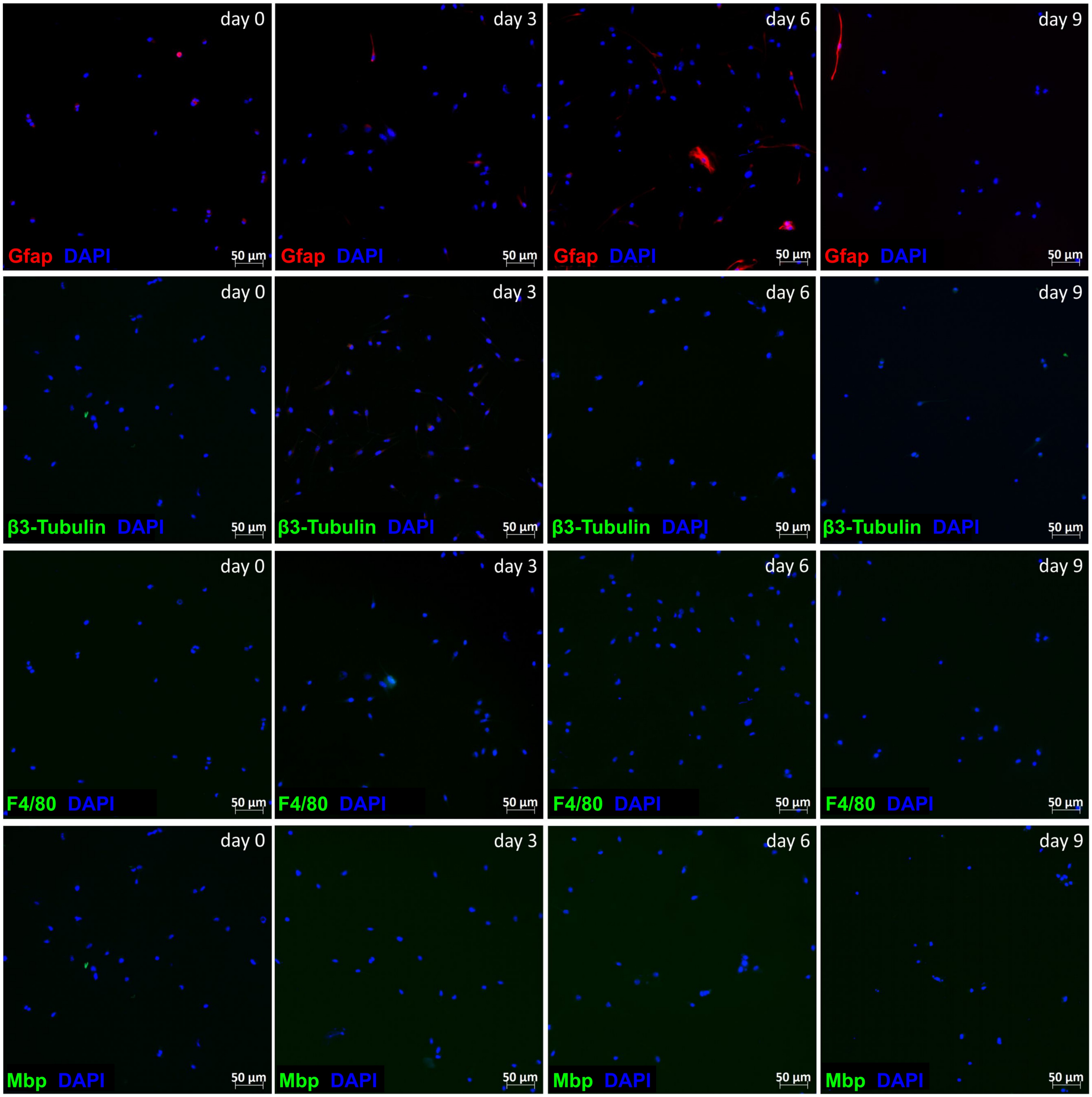
day 0
day 3
day 6
day 9
Gfap DAPI
β3-Tubulin DAPI
F4/80 DAPI
Mbp DAPI
50 µm

Figure S2

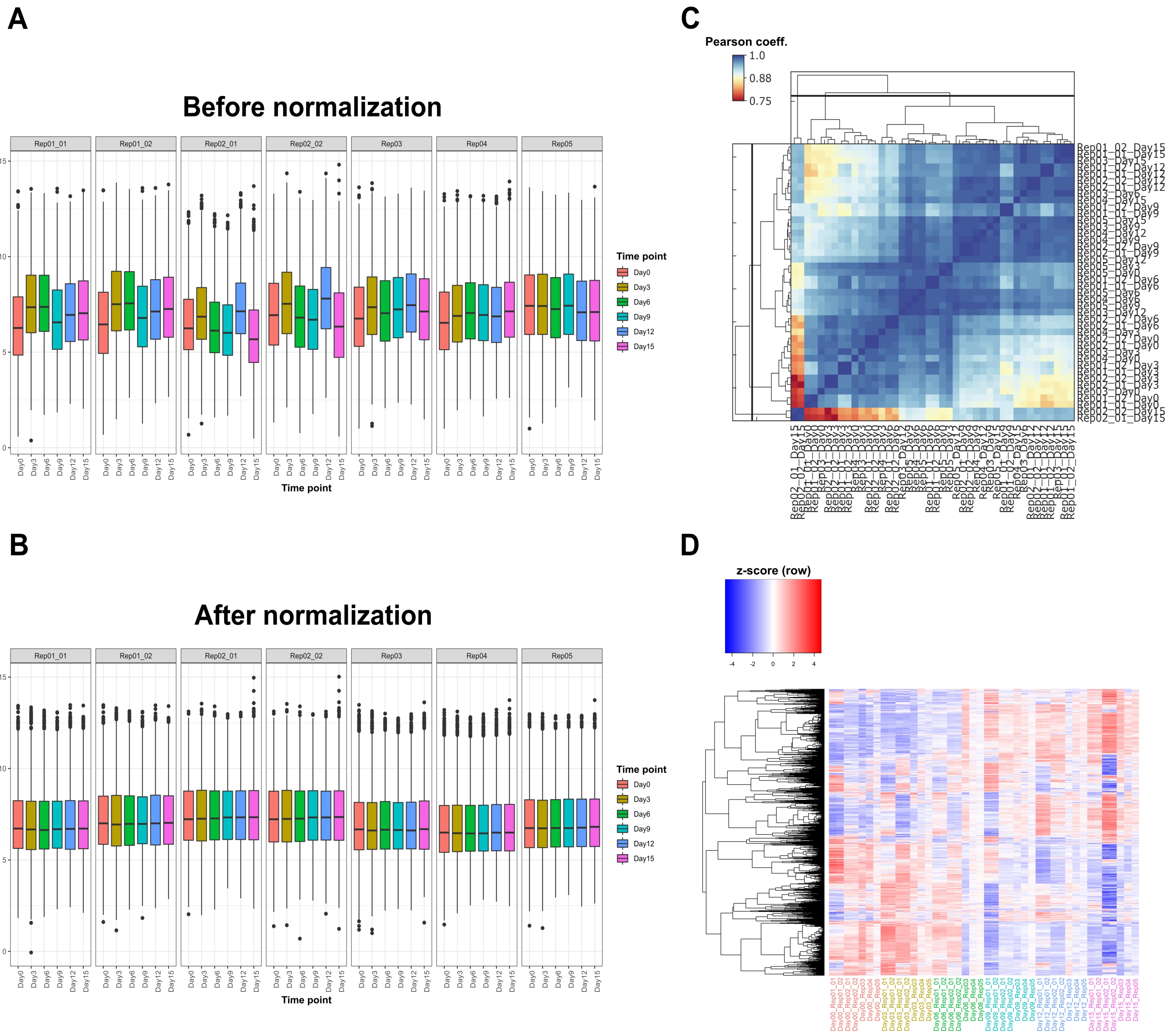
A
Before normalization
Rep01_01
Rep01_02
Rep02_01
Rep02_02
Rep03
Rep04
Rep05
Log2 abundance
Time point
Day0
Day3
Day6
Day9
Day12
Day15
B
After normalization
C
Pearson coeff.
1.0
0.88
0.75
D
z-score (row)
-4
-2
0
2
4

Figure S3

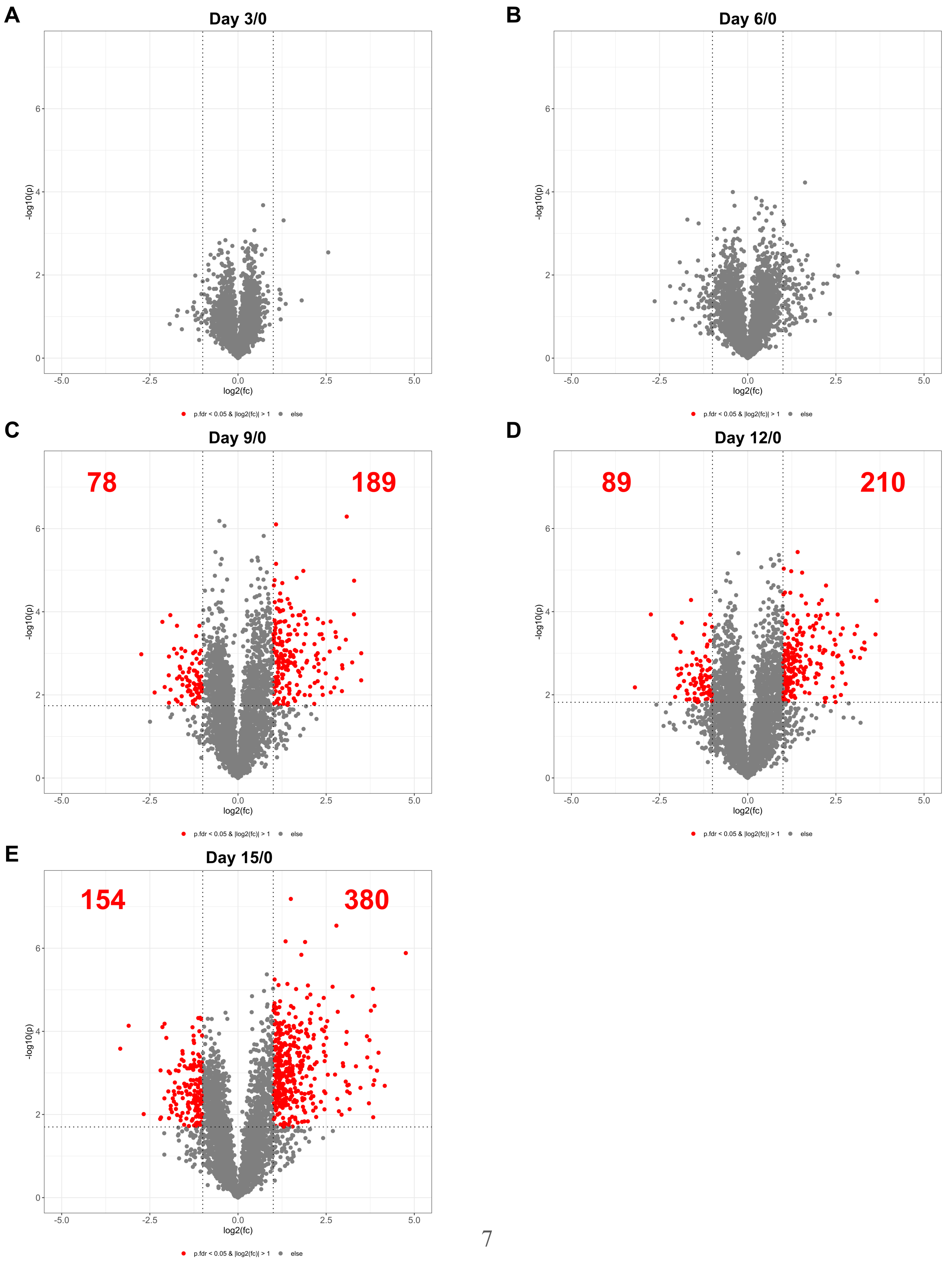
A
Day 3/0
B
Day 6/0
C
Day 9/0
78
189
D
Day 12/0
89
210
E
Day 15/0
154
380
-log10(p)
log2(fc)
p.fdr < 0.05 & |log2(fc)| > 1
else

Figure S4

A

Biological Process

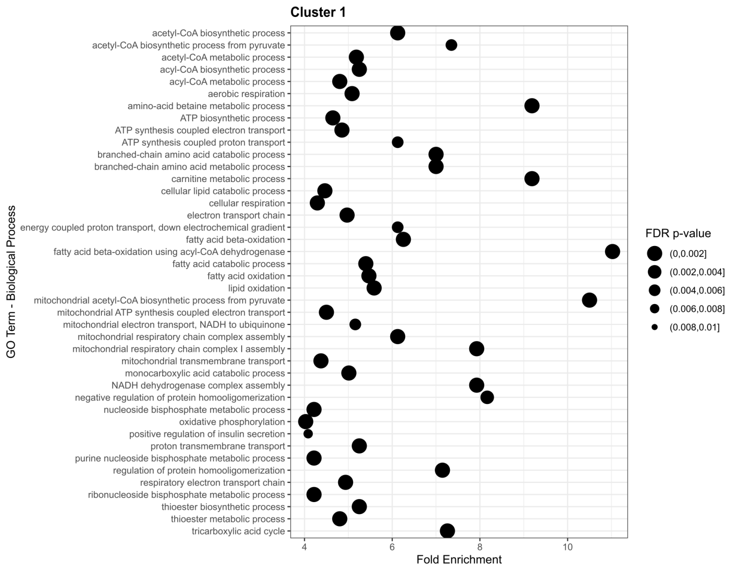

Cellular Compartment

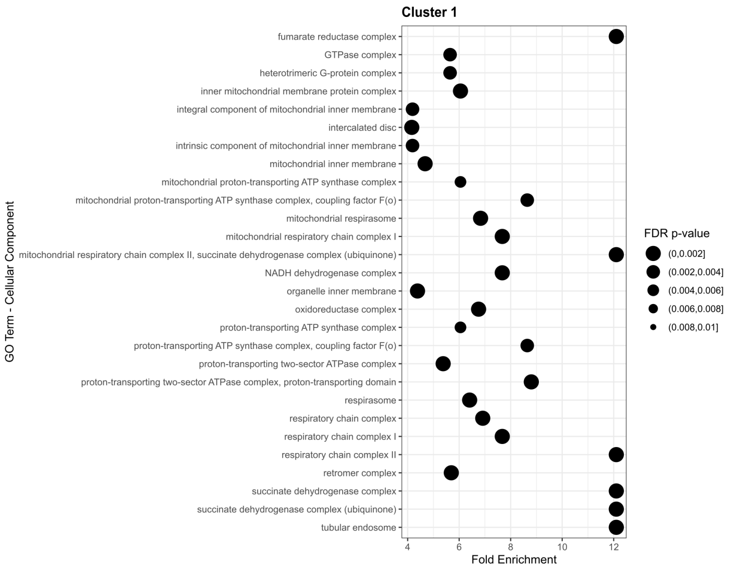

B

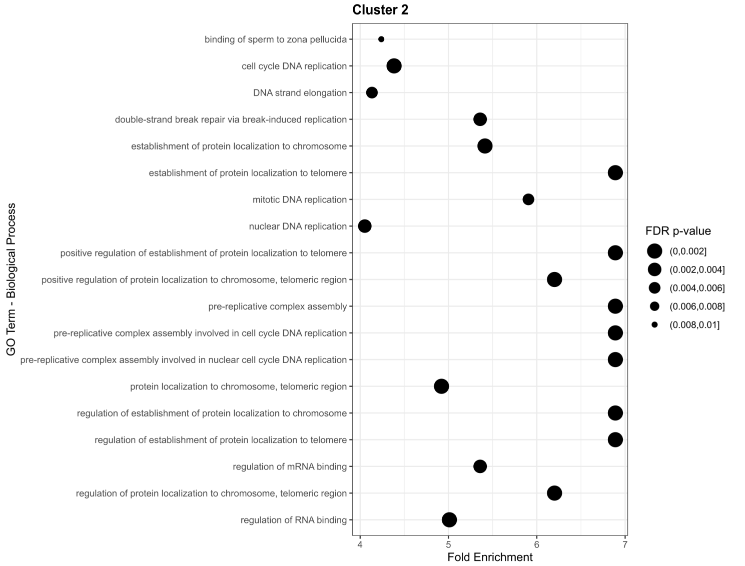

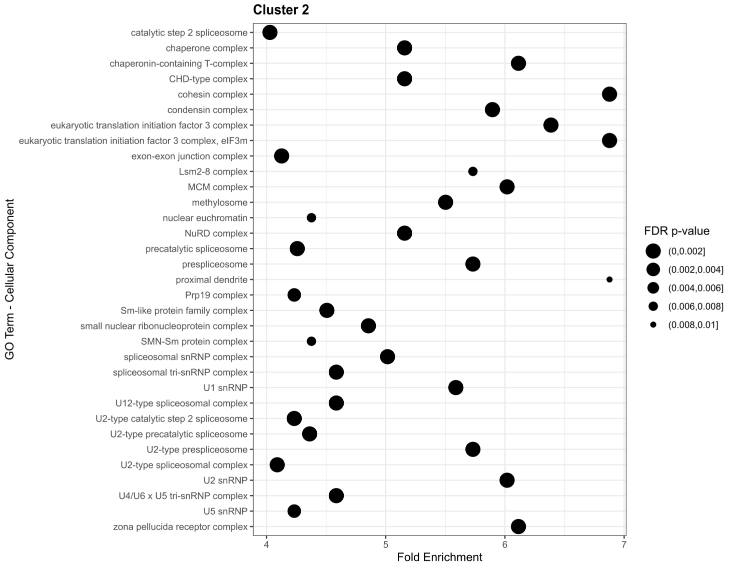

C

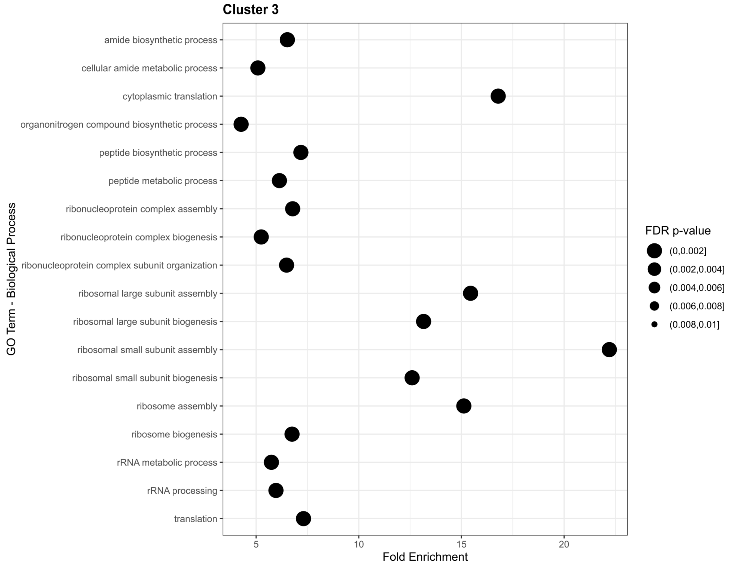

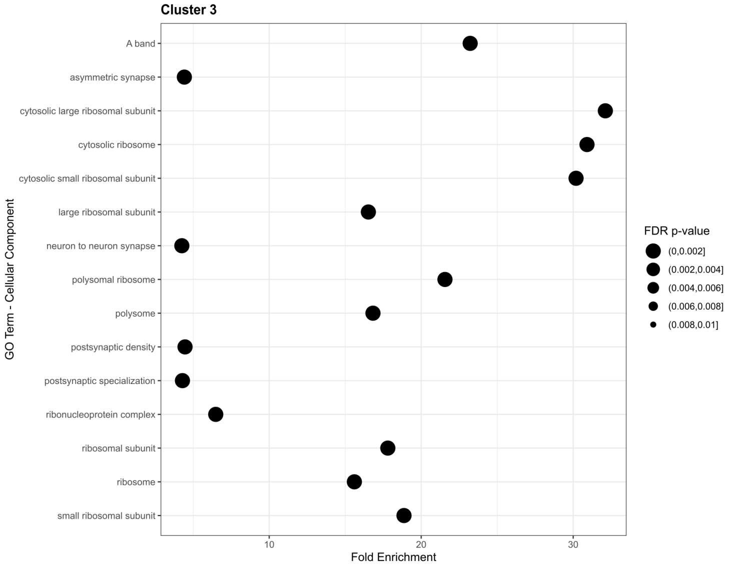

Figure S4

D

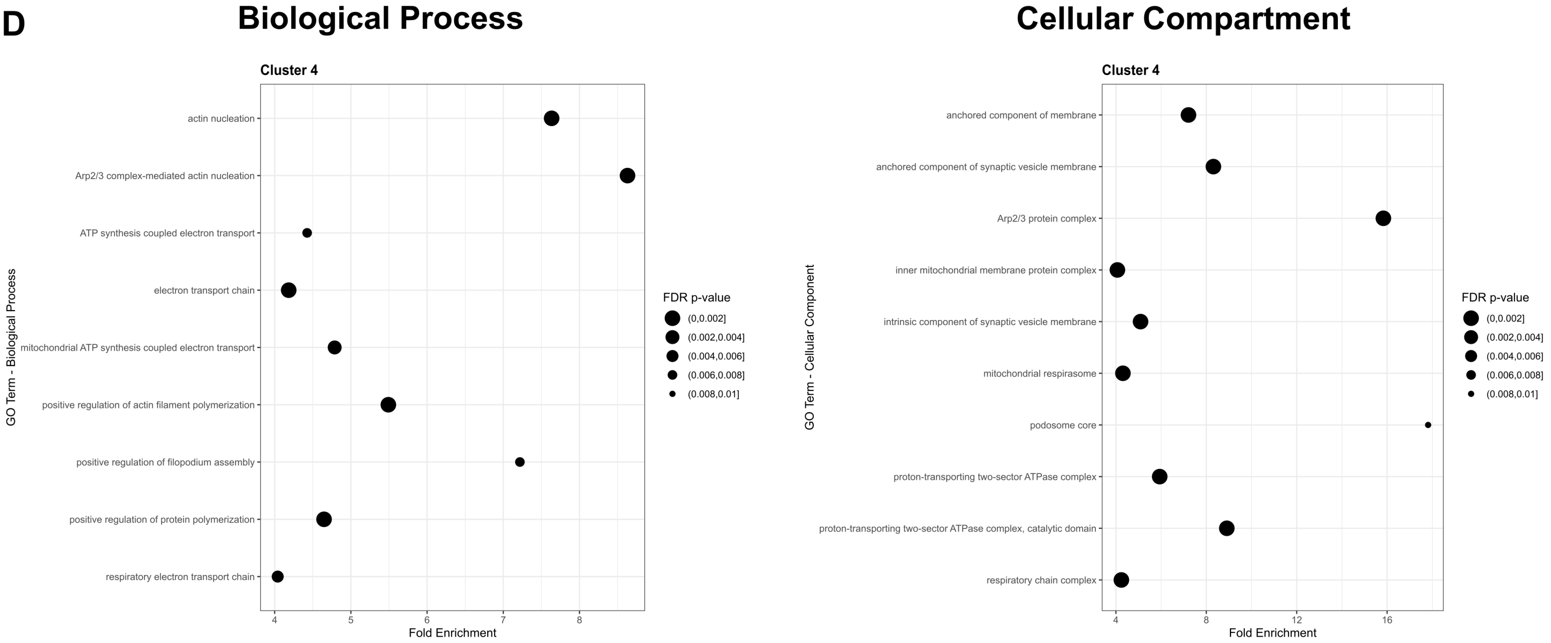

Biological Process
Cluster 4
actin nucleation
Arp2/3 complex-mediated actin nucleation
ATP synthesis coupled electron transport
electron transport chain
mitochondrial ATP synthesis coupled electron transport
positive regulation of actin filament polymerization
positive regulation of filopodium assembly
positive regulation of protein polymerization
respiratory electron transport chain
GO Term - Biological Process
Fold Enrichment
FDR p-value
(0,0.002]
(0.002,0.004]
(0.004,0.006]
(0.006,0.008]
(0.008,0.01]
Cellular Compartment
Cluster 4
anchored component of membrane
anchored component of synaptic vesicle membrane
Arp2/3 protein complex
inner mitochondrial membrane protein complex
intrinsic component of synaptic vesicle membrane
mitochondrial respirasome
podosome core
proton-transporting two-sector ATPase complex
proton-transporting two-sector ATPase complex, catalytic domain
respiratory chain complex
GO Term - Cellular Component
Fold Enrichment
FDR p-value
(0,0.002]
(0.002,0.004]
(0.004,0.006]
(0.006,0.008]
(0.008,0.01]

E

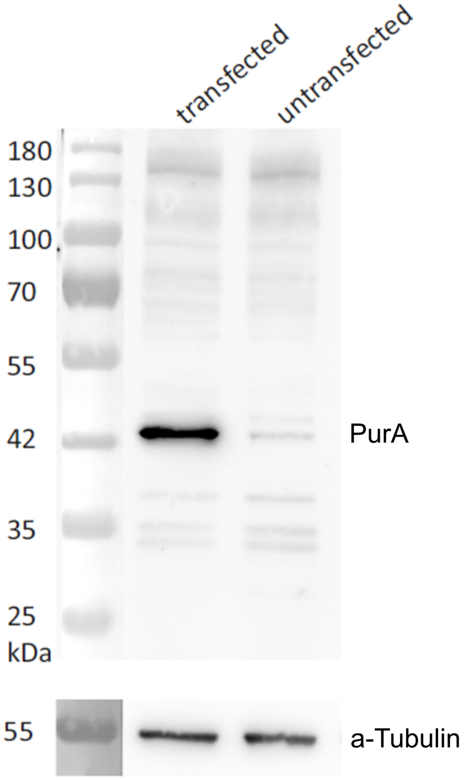

transfected
untransfected
180
130
100
70
55
42
35
25
kDa
PurA
55
a-Tubulin

Figure S5

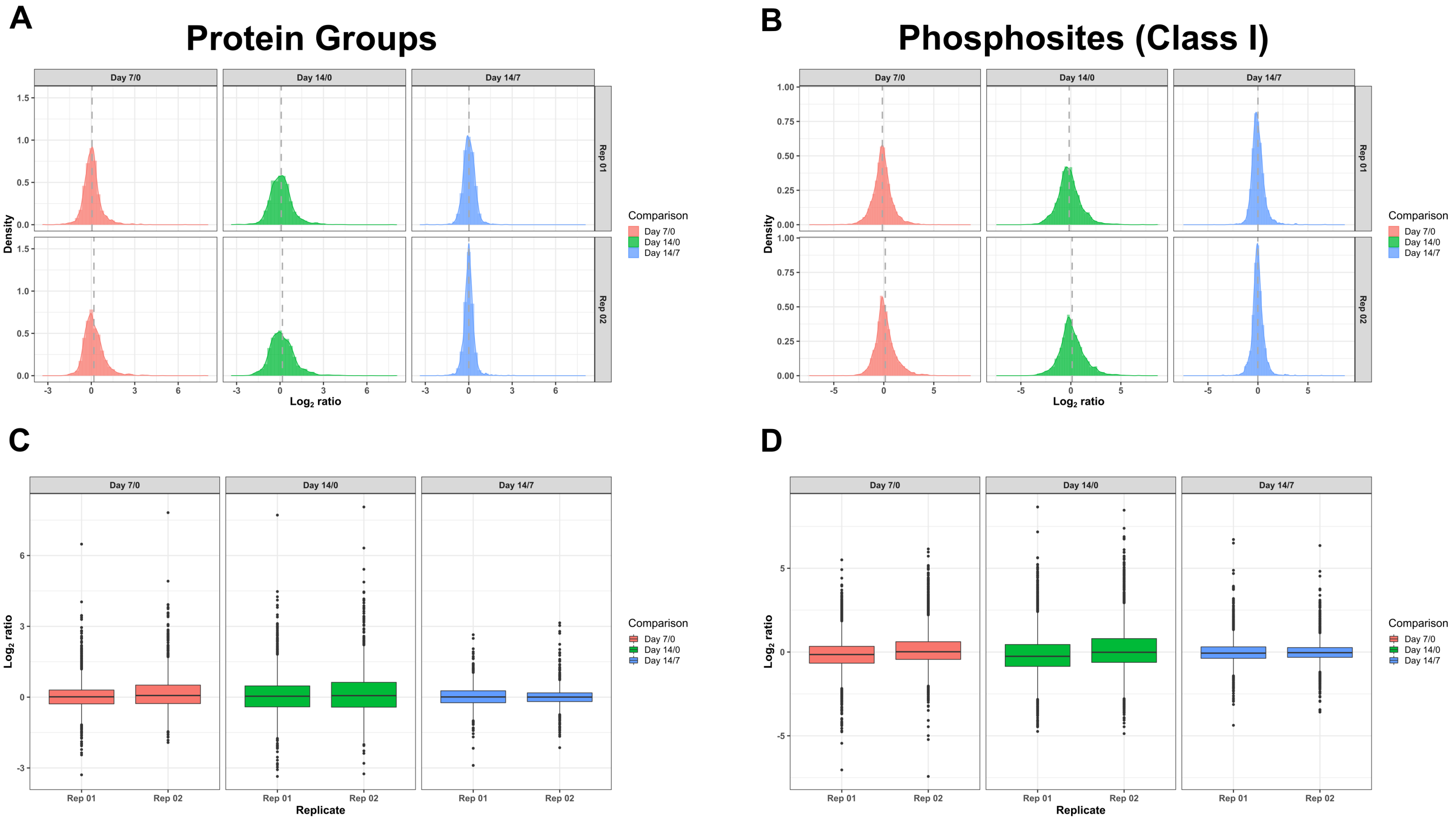
A
Protein Groups
B
Phosphosites (Class I)
Day 7/0
Day 14/0
Day 14/7
Rep 01
Rep 02
Density
Log2 ratio
Comparison
Day 7/0
Day 14/0
Day 14/7
C
D
Replicate

Figure S6

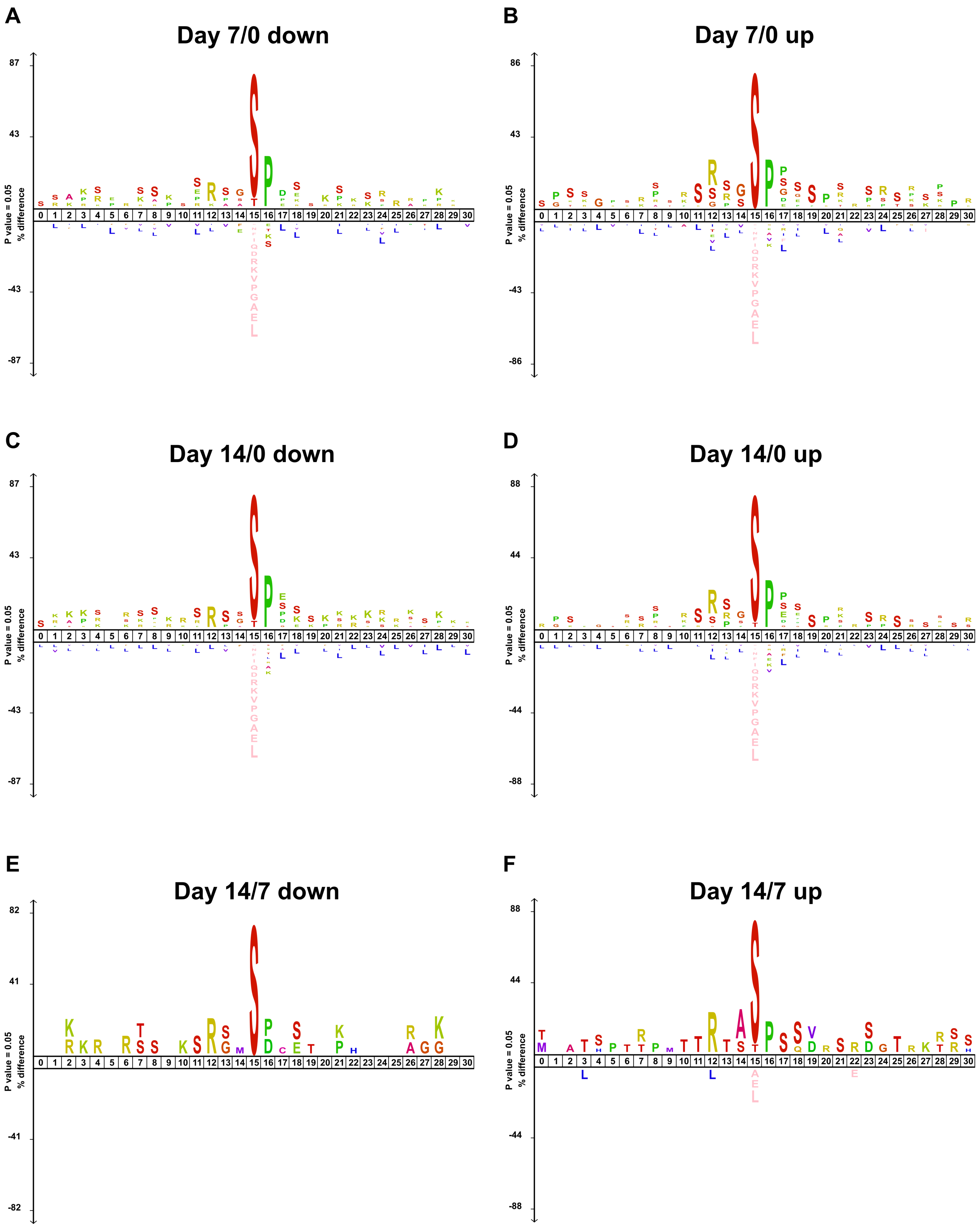

A
Day 7/0 down
B
Day 7/0 up
C
Day 14/0 down
D
Day 14/0 up
E
Day 14/7 down
F
Day 14/7 up
P value = 0.05
% difference
87
43
-43
-87
86
-86
88
44
-44
-88
82
41
-41
-82

Figure S7

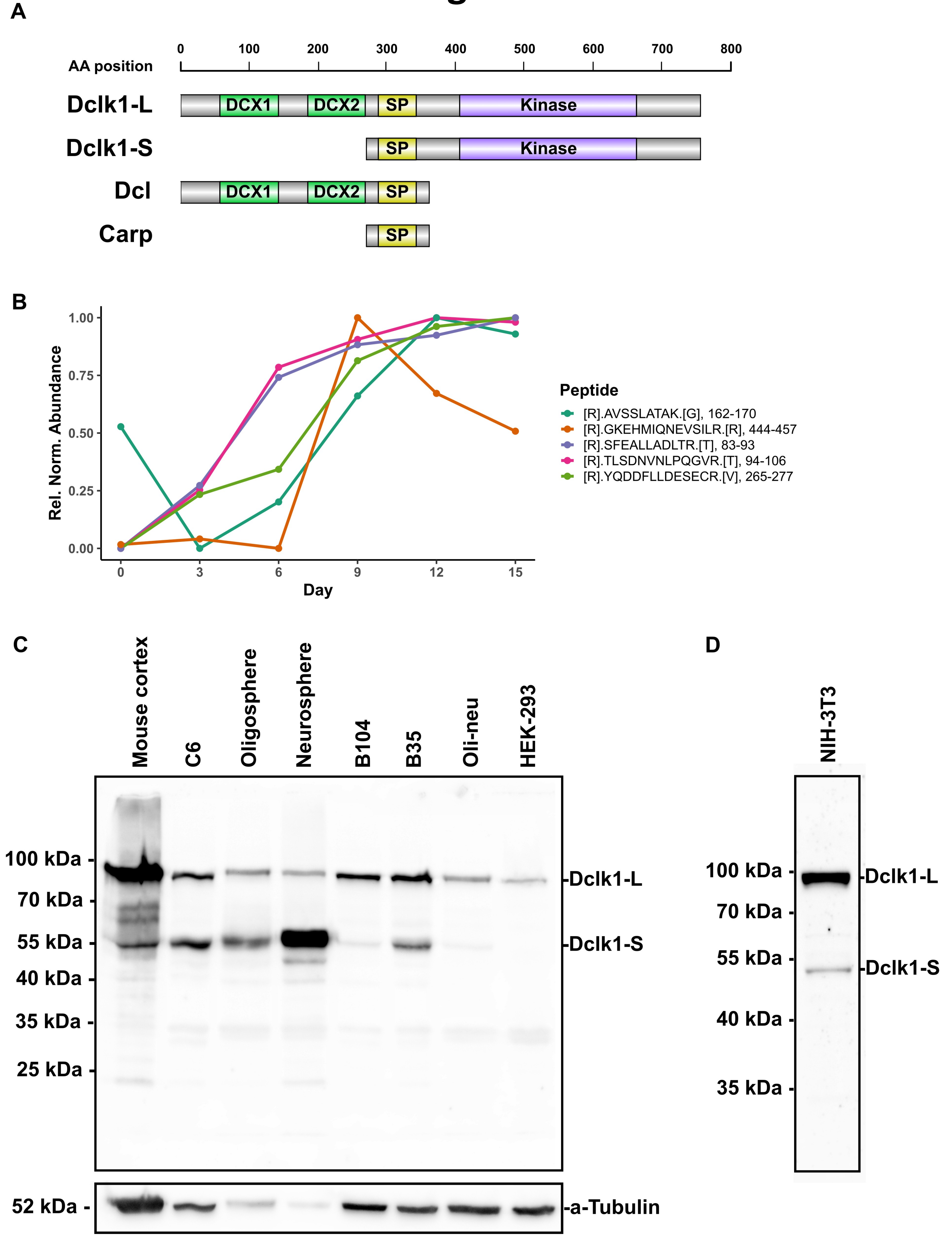
A
AA position
0
100
200
300
400
500
600
700
800
Dclk1-L
DCX1
DCX2
SP
Kinase
Dclk1-S
SP
Kinase
Dcl
DCX1
DCX2
SP
Carp
SP
B
Rel. Norm. Abundance
1.00
0.75
0.50
0.25
0.00
0
3
6
9
12
15
Day
Peptide
[R].AVSSLATAK.[G], 162-170
[R].GKEHMIQNEVSILR.[R], 444-457
[R].SFEALLADLTR.[T], 83-93
[R].TLSDNVNLPQGVR.[T], 94-106
[R].YQDDFLLDESECR.[V], 265-277
C
Mouse cortex
C6
Oligosphere
Neurosphere
B104
B35
Oli-neu
HEK-293
100 kDa
70 kDa
55 kDa
40 kDa
35 kDa
25 kDa
Dclk1-L
Dclk1-S
52 kDa
a-Tubulin
D
NIH-3T3
100 kDa
70 kDa
55 kDa
40 kDa
35 kDa
Dclk1-L
Dclk1-S

Figure S8

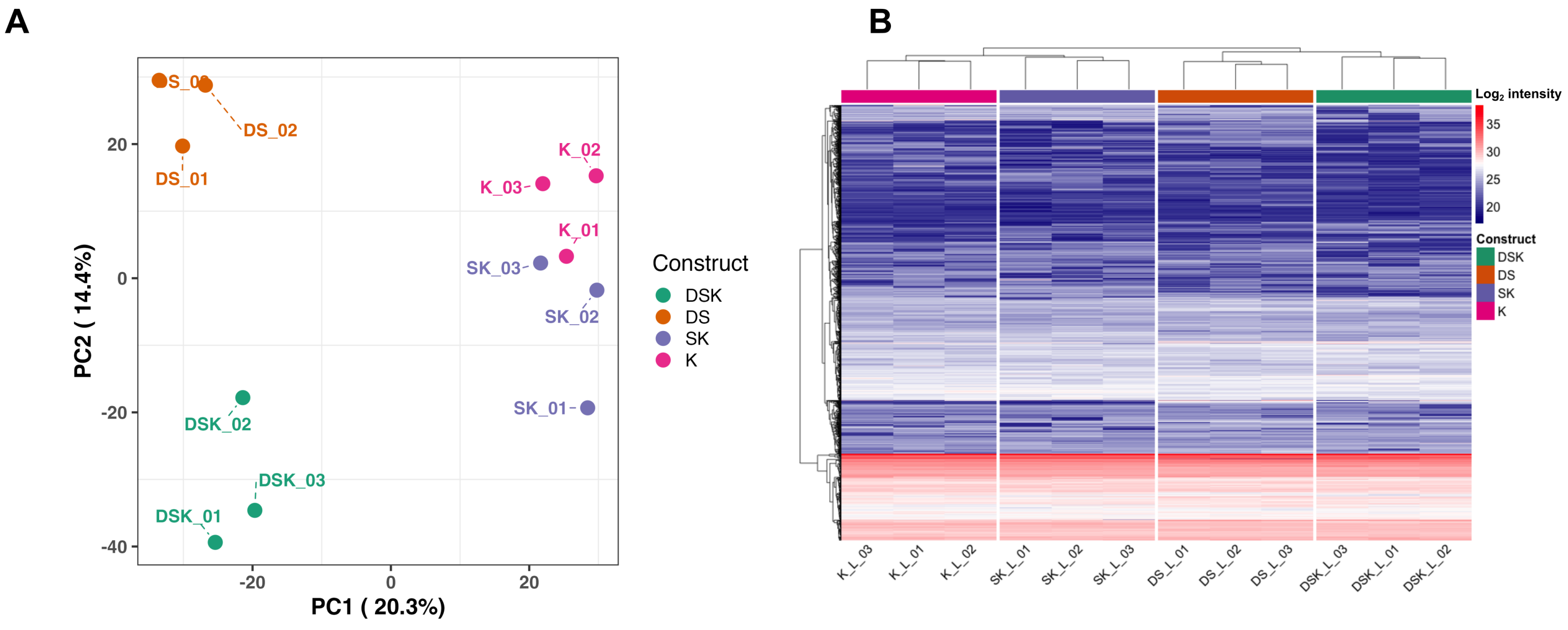

A
B
DS_02
DS_01
K_02
K_03
K_01
SK_03
SK_02
SK_01
DSK_02
DSK_03
DSK_01
PC1 (20.3%)
PC2 (14.4%)
Construct
DSK
DS
SK
K
Log2 intensity
35
30
25
20
K_L_03
K_L_01
K_L_02
SK_L_01
SK_L_02
SK_L_03
DS_L_01
DS_L_02
DS_L_03
DSK_L_03
DSK_L_01
DSK_L_02

Figure S9

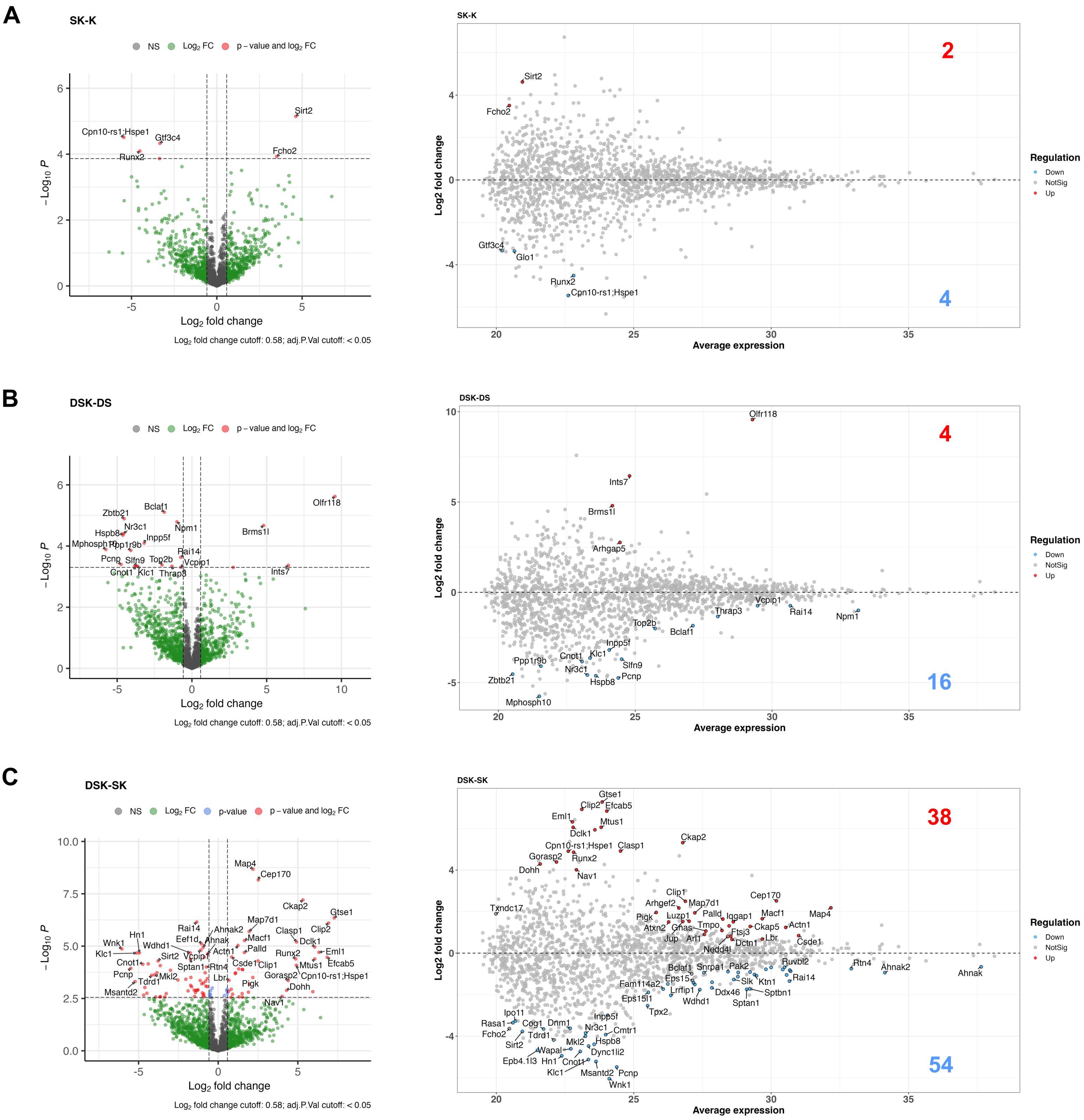
A
SK-K
NS
Log2 FC
p – value and log2 FC
−Log10 P
Log2 fold change
Log2 fold change cutoff: 0.58; adj.P.Val cutoff: < 0.05
Sirt2
Cpn10-rs1;Hspe1
Gtf3c4
Runx2
Fcho2
SK-K
2
4
Glo1
Log2 fold change
Average expression
Regulation
Down
NotSig
Up
B
DSK-DS
Olfr118
Ints7
Brms1l
Arhgap5
Vcpip1
Thrap3
Rai14
Npm1
Top2b
Bclaf1
Inpp5f
Cnot1
Klc1
Ppp1r9b
Nr3c1
Slfn9
Pcnp
Hspb8
Zbtb21
Mphosph10
Nr3c1
Inpp5f
Slfn9
4
16
C
DSK-SK
p-value
38
54
Gtse1
Clip2
Efcab5
Eml1
Mtus1
Dclk1
Ckap2
Cpn10-rs1;Hspe1
Clasp1
Gorasp2
Runx2
Dohh
Nav1
Map4
Cep170
Map7d1
Ahnak
Ahnak2
Rtn4
Rai14
Macf1
Actn1
Csde1
Txndc17
Wdhd1
Sptan1
Sptbn1
Ipo11
Rasa1
Fcho2
Sirt2
Tdrd1
Wnk1
Klc1
Msantd2
Pcnp
Mkl2
Hn1
Epb4.1l3
Wapal
Dync1li2
Cmtr1
Tpx2
Eps15l1
Fam114a2
Ruvbl2

Figure S9

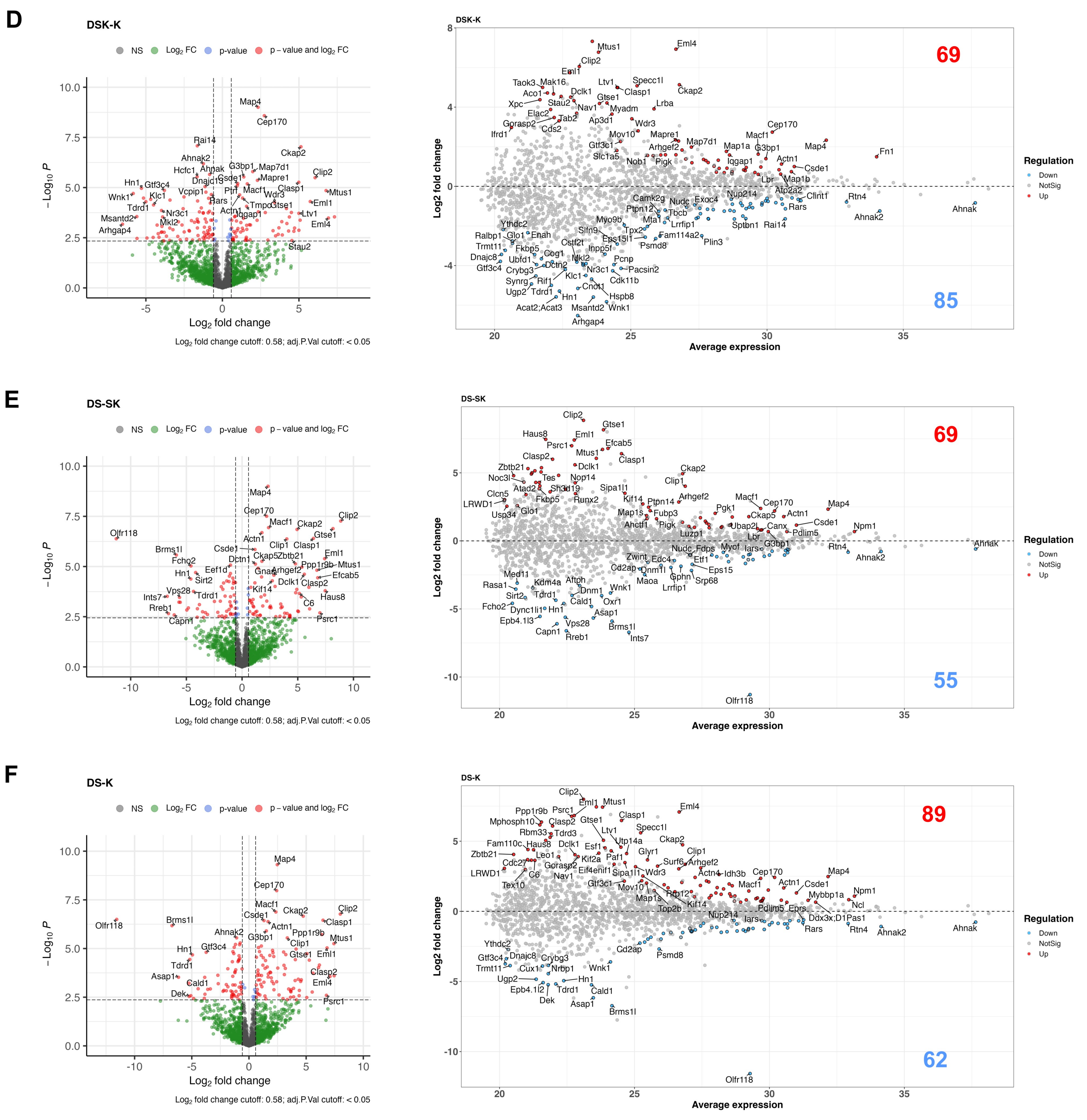
D
DSK-K
NS
Log2 FC
p-value
p – value and log2 FC
Log2 fold change
−Log10 P
Log2 fold change cutoff: 0.58; adj.P.Val cutoff: < 0.05
DSK-K
69
85
Log2 fold change
Average expression
Regulation
Down
NotSig
Up
E
DS-SK
NS
Log2 FC
p-value
p – value and log2 FC
Log2 fold change
−Log10 P
Log2 fold change cutoff: 0.58; adj.P.Val cutoff: < 0.05
DS-SK
69
55
Log2 fold change
Average expression
Regulation
Down
NotSig
Up
F
DS-K
NS
Log2 FC
p-value
p – value and log2 FC
Log2 fold change
−Log10 P
Log2 fold change cutoff: 0.58; adj.P.Val cutoff: < 0.05
DS-K
89
62
Log2 fold change
Average expression
Regulation
Down
NotSig
Up

Figure S9

G

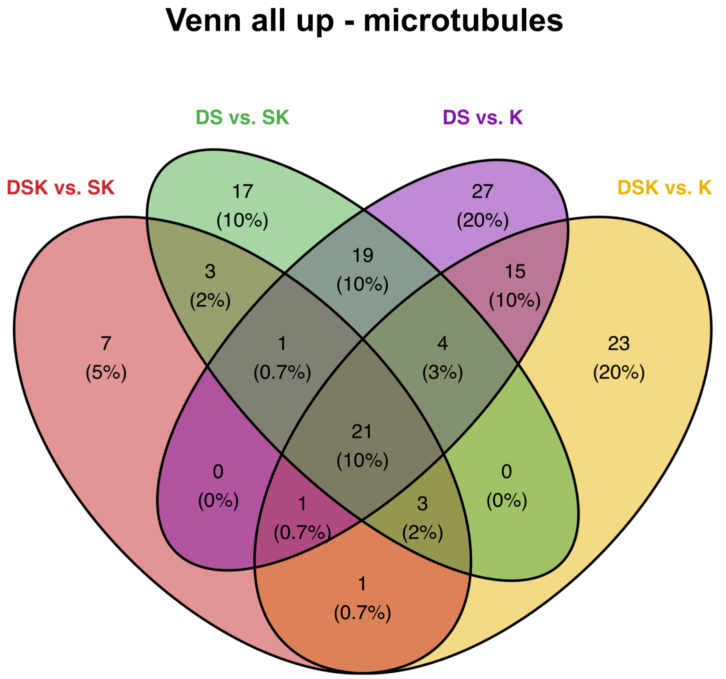

Venn all up - microtubules
DS vs. SK
DS vs. K
DSK vs. SK
DSK vs. K
17
(10%)
27
(20%)
3
(2%)
19
(10%)
15
(10%)
7
(5%)
1
(0.7%)
4
(3%)
23
(20%)
21
(10%)
0
(0%)
0
(0%)
1
(0.7%)
3
(2%)
1
(0.7%)

H

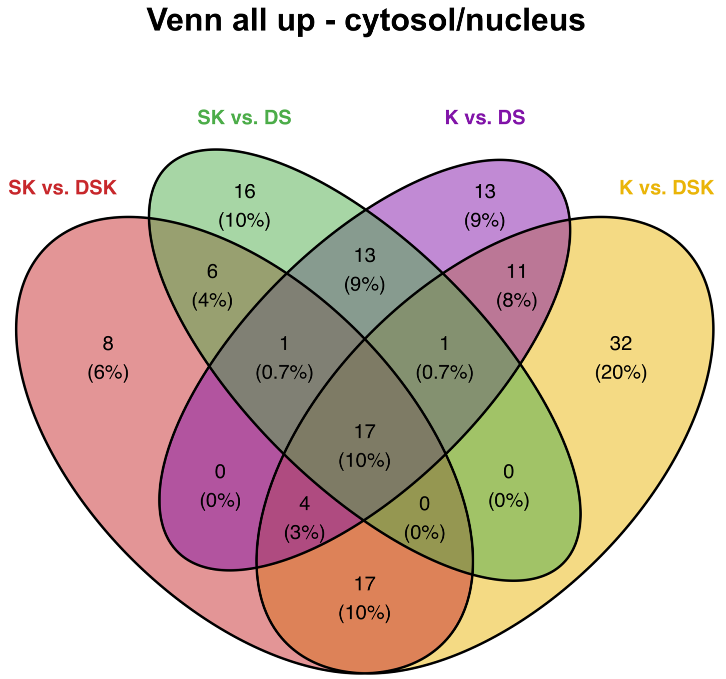

Venn all up - cytosol/nucleus
SK vs. DS
K vs. DS
SK vs. DSK
K vs. DSK
16
(10%)
13
(9%)
6
(4%)
13
(9%)
11
(8%)
8
(6%)
1
(0.7%)
1
(0.7%)
32
(20%)
17
(10%)
0
(0%)
0
(0%)
4
(3%)
0
(0%)
17
(10%)

Figure S10

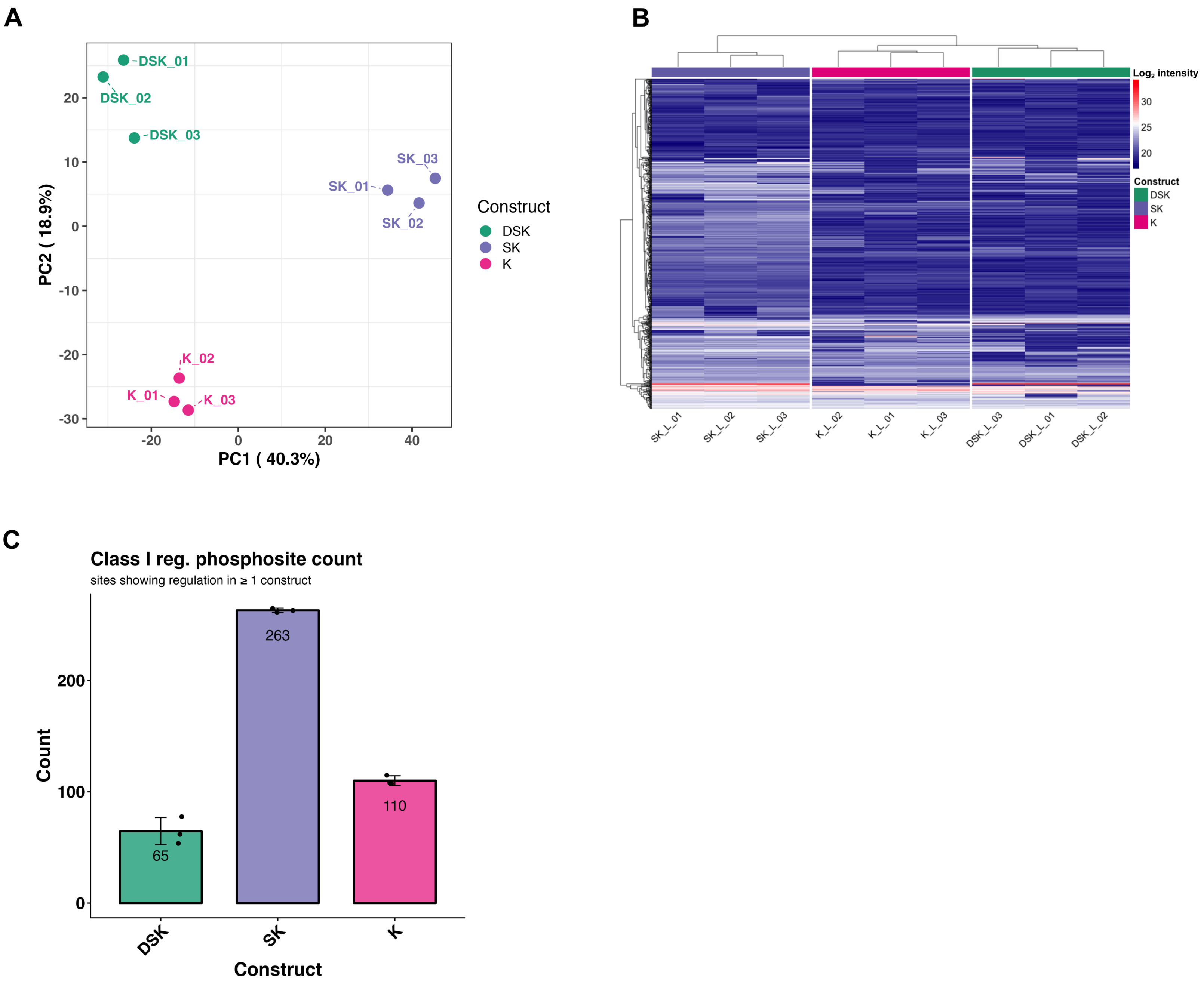
A
DSK_01
DSK_02
DSK_03
SK_03
SK_01
SK_02
K_02
K_01
K_03
PC2 (18.9%)
PC1 (40.3%)
Construct
DSK
SK
K
B
Log2 intensity
30
25
20
Construct
DSK
SK
K
SK_L_01
SK_L_02
SK_L_03
K_L_02
K_L_01
K_L_03
DSK_L_03
DSK_L_01
DSK_L_02
C
Class I reg. phosphosite count
sites showing regulation in ≥ 1 construct
Count
65
263
110
DSK
SK
K
Construct

Figure S11

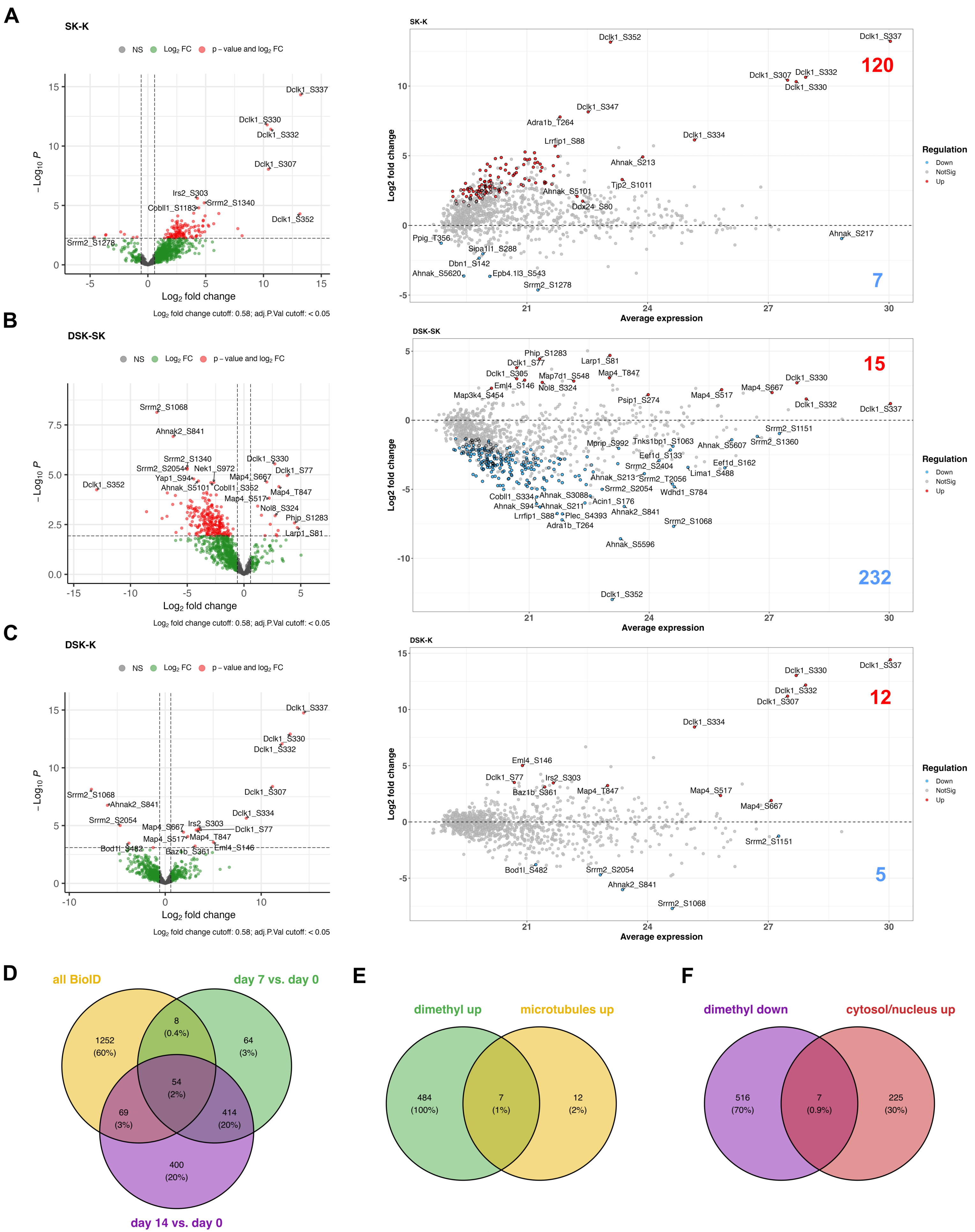

A
SK-K
NS
Log2 FC
p – value and log2 FC
Dclk1_S337
Dclk1_S330
Dclk1_S332
Dclk1_S307
Irs2_S303
Srrm2_S1340
Cobll1_S1183
Dclk1_S352
Srrm2_S1278
Log2 fold change
−Log10 P
Log2 fold change cutoff: 0.58; adj.P.Val cutoff: < 0.05
SK-K
Dclk1_S352
Dclk1_S337
120
Dclk1_S307
Dclk1_S332
Dclk1_S330
Dclk1_S347
Adra1b_T264
Lrrfip1_S88
Dclk1_S334
Ahnak_S213
Tjp2_S1011
Ahnak_S5101
Ddx24_S80
Ppig_T356
Ahnak_S217
Sipa1l1_S288
Dbn1_S142
Ahnak_S5620
Epb4.1l3_S543
Srrm2_S1278
7
Log2 fold change
Average expression
Regulation
Down
NotSig
Up
B
DSK-SK
NS
Log2 FC
p – value and log2 FC
Srrm2_S1068
Ahnak2_S841
Srrm2_S1340
Srrm2_S2054
Nek1_S972
Dclk1_S330
Dclk1_S77
Yap1_S94
Map4_S667
Dclk1_S352
Ahnak_S5101
Cobll1_S352
Map4_T847
Map4_S517
Nol8_S324
Phip_S1283
Larp1_S81
Log2 fold change
−Log10 P
Log2 fold change cutoff: 0.58; adj.P.Val cutoff: < 0.05
DSK-SK
Phip_S1283
Dclk1_S77
Larp1_S81
15
Dclk1_S305
Map7d1_S548
Map4_T847
Eml4_S146
Nol8_S324
Map3k4_S454
Psip1_S274
Map4_S517
Map4_S667
Dclk1_S330
Dclk1_S332
Dclk1_S337
Srrm2_S1151
Mprip_S992
Tnks1bp1_S1063
Ahnak_S5607
Srrm2_S1360
Eef1d_S133
Eef1d_S162
Srrm2_S2404
Lima1_S488
Ahnak_S213
Srrm2_T2056
Srrm2_S2054
Wdhd1_S784
Cobll1_S334
Ahnak_S3088
Acin1_S176
Ahnak_S94
Ahnak_S211
Ahnak2_S841
Lrrfip1_S88
Plec_S4393
Srrm2_S1068
Adra1b_T264
Ahnak_S5596
232
Dclk1_S352
Log2 fold change
Average expression
Regulation
Down
NotSig
Up
C
DSK-K
NS
Log2 FC
p – value and log2 FC
Dclk1_S337
Dclk1_S330
Dclk1_S332
Srrm2_S1068
Dclk1_S307
Ahnak2_S841
Dclk1_S334
Srrm2_S2054
Irs2_S303
Map4_S667
Dclk1_S77
Map4_S517
Map4_T847
Bod1l_S482
Baz1b_S361
Eml4_S146
Log2 fold change
−Log10 P
Log2 fold change cutoff: 0.58; adj.P.Val cutoff: < 0.05
DSK-K
Dclk1_S337
Dclk1_S330
Dclk1_S332
Dclk1_S307
12
Dclk1_S334
Eml4_S146
Dclk1_S77
Irs2_S303
Baz1b_S361
Map4_T847
Map4_S517
Map4_S667
Srrm2_S1151
Bod1l_S482
Srrm2_S2054
Ahnak2_S841
Srrm2_S1068
5
Log2 fold change
Average expression
Regulation
Down
NotSig
Up
D
all BioID
day 7 vs. day 0
1252 (60%)
8 (0.4%)
64 (3%)
54 (2%)
69 (3%)
414 (20%)
400 (20%)
day 14 vs. day 0
E
dimethyl up
microtubules up
484 (100%)
7 (1%)
12 (2%)
F
dimethyl down
cytosol/nucleus up
516 (70%)
7 (0.9%)
225 (30%)

Figure S12

A

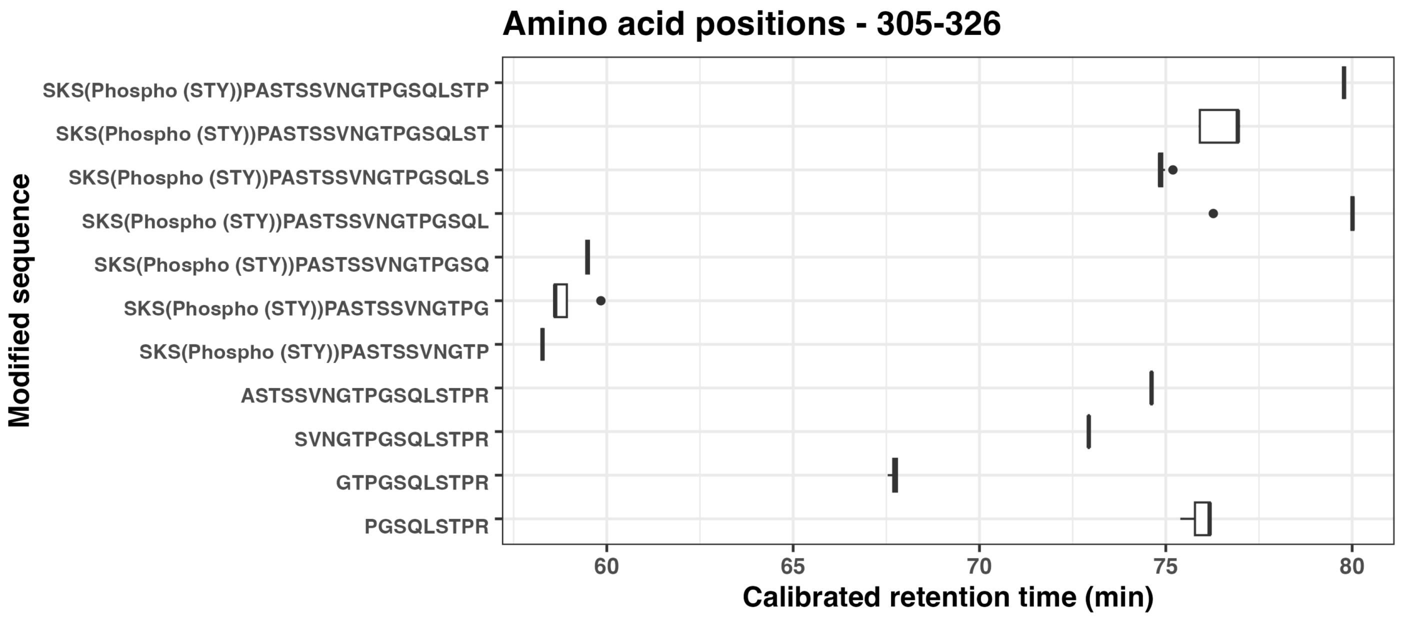

B

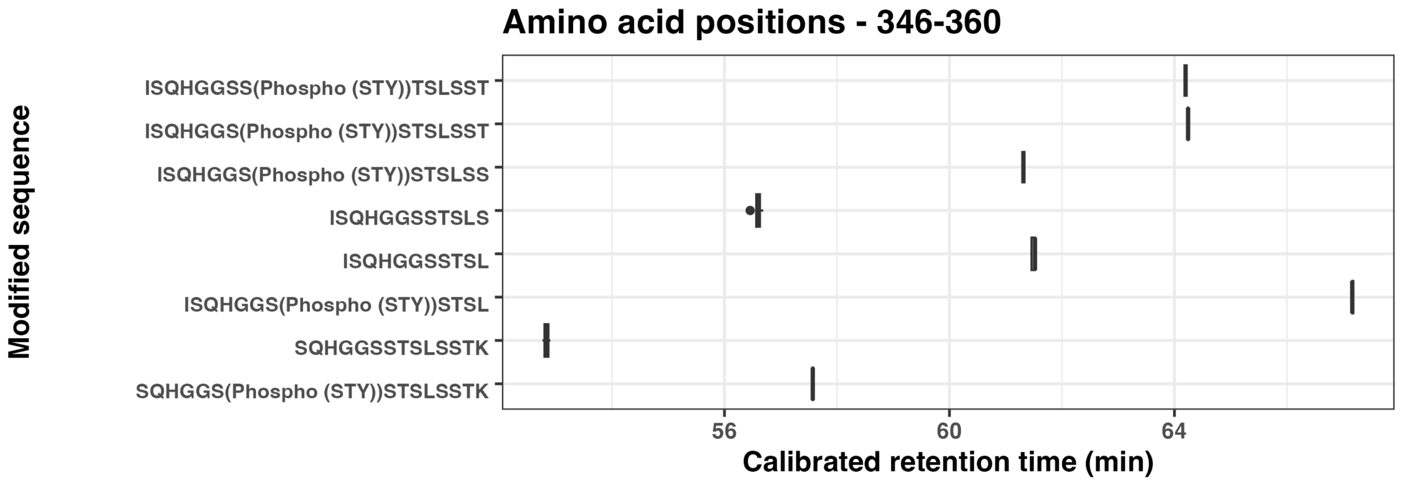

Uncropped Western Blots and Agarose Gels

Figure 2

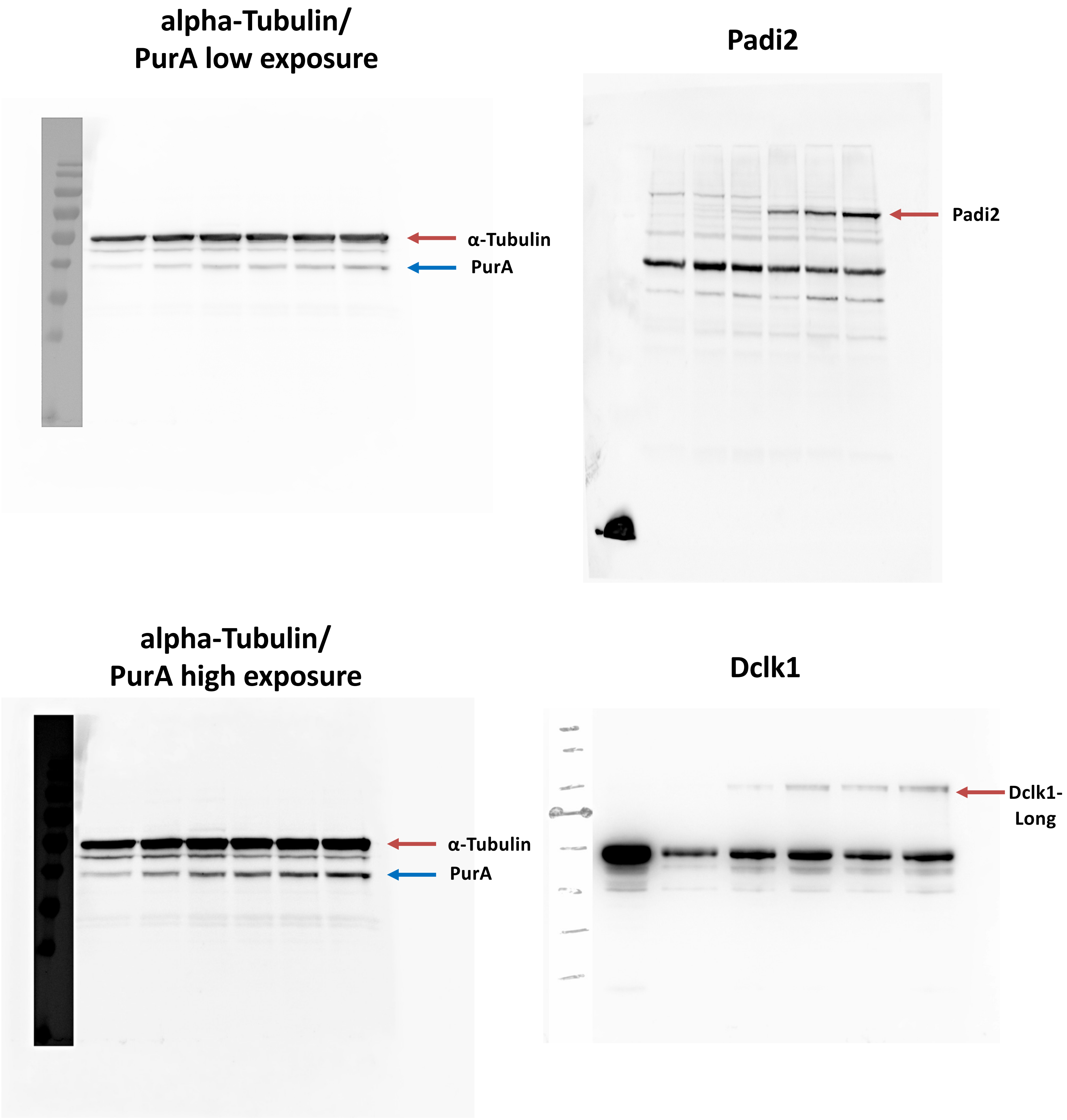
alpha-Tubulin/
PurA low exposure
α-Tubulin
PurA
Padi2
Padi2
alpha-Tubulin/
PurA high exposure
α-Tubulin
PurA
Dclk1
Dclk1-
Long

Figure 4

Replicate 1 **Replicate 2**

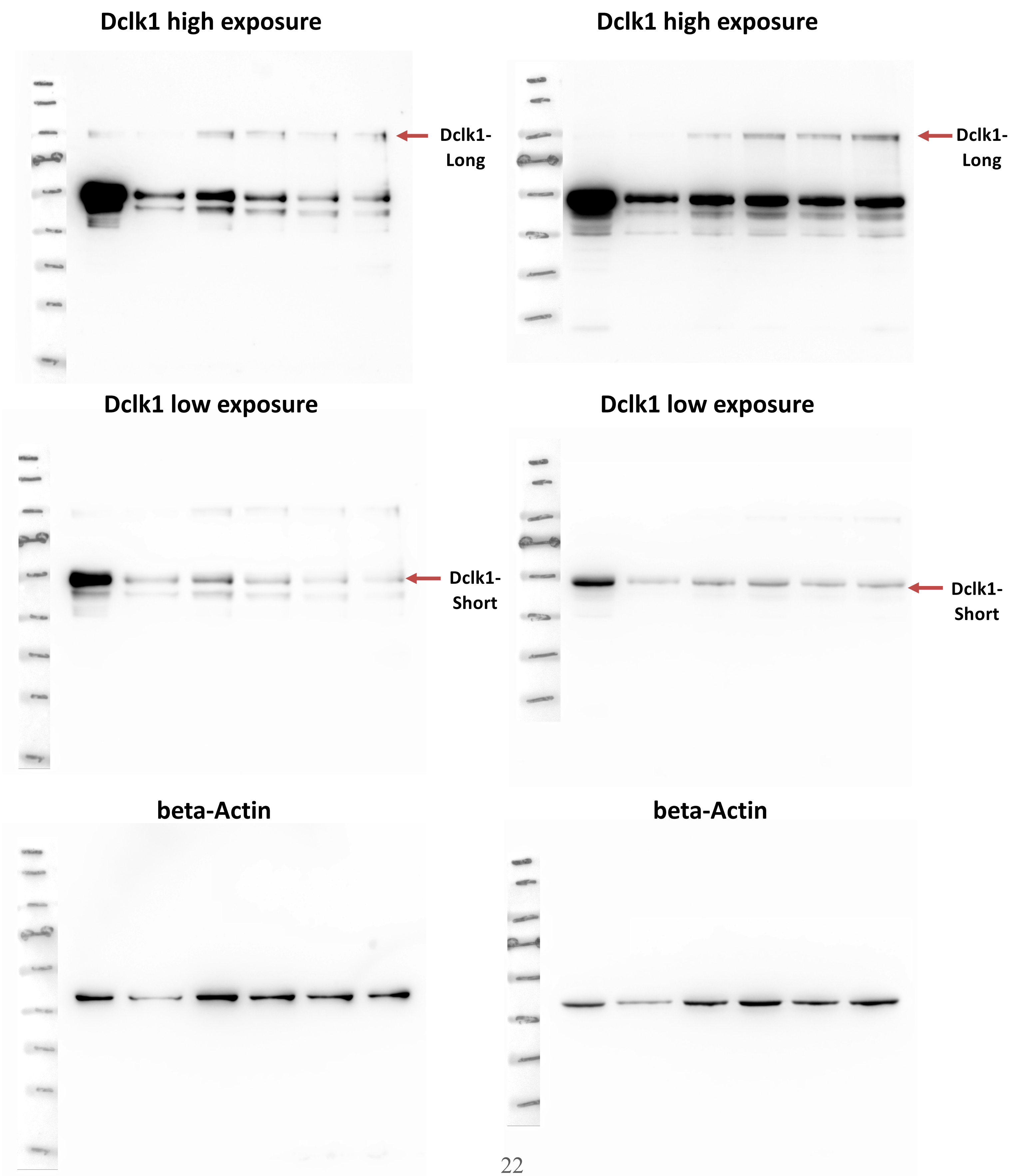

Figure 4

Replicate 3

Dclk1 high exposure

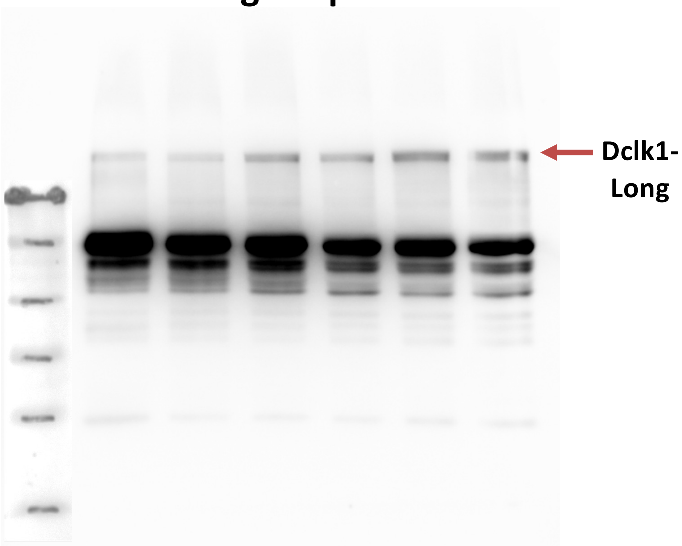

Dclk1-Long

Dclk1 low exposure

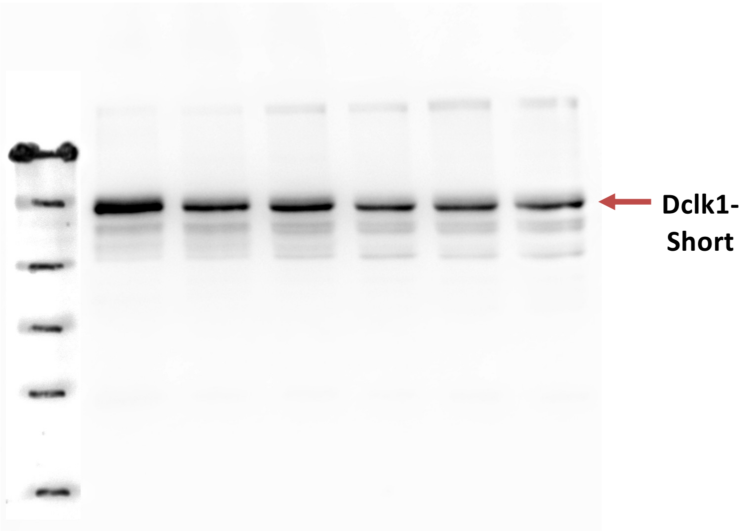

Dclk1-Short

beta-Actin

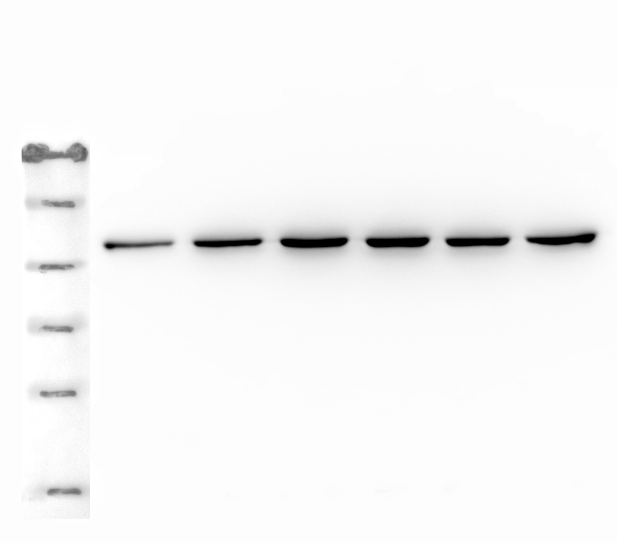

Replicate 4

Dclk1 high exposure

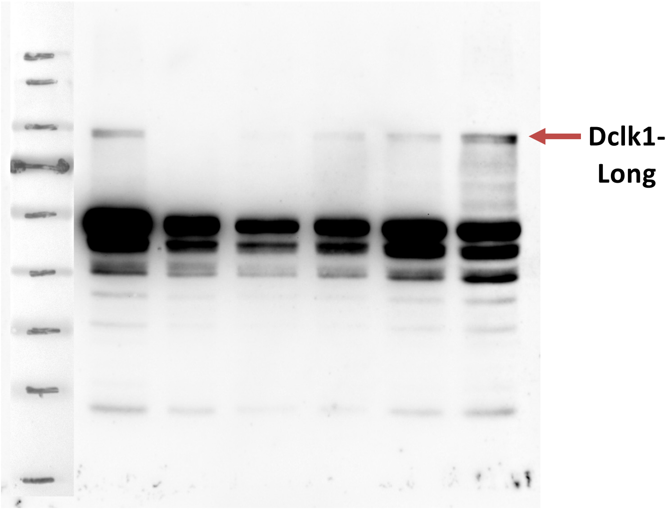

Dclk1-Long

Dclk1 low exposure

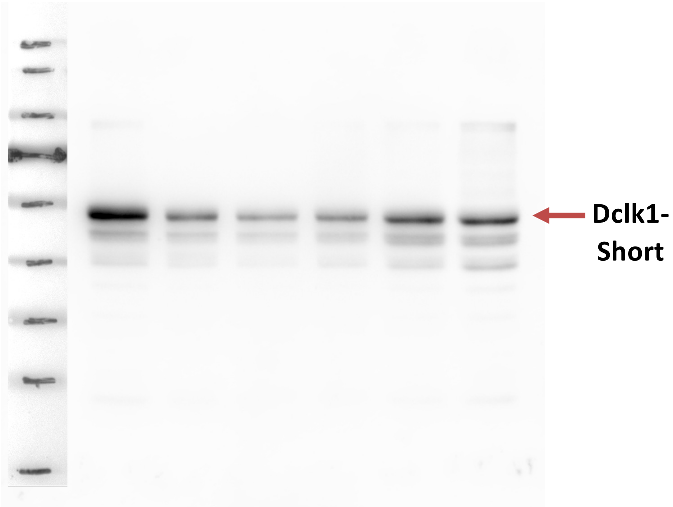

Dclk1-Short

beta-Actin

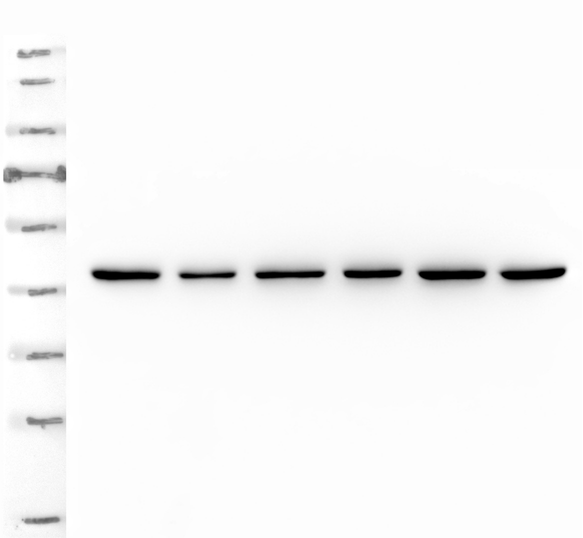

Figure 4

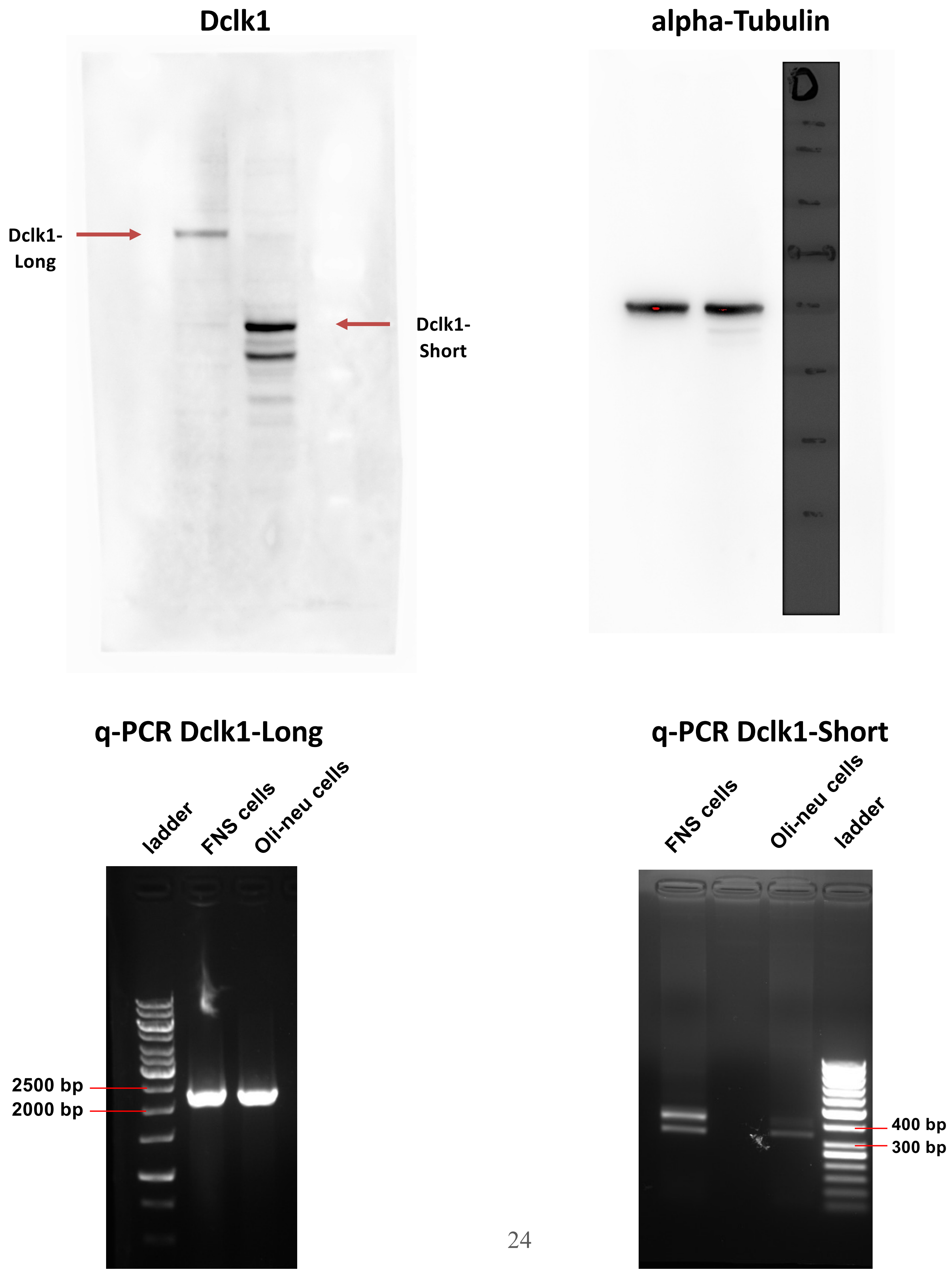
Dclk1
alpha-Tubulin
Dclk1-
Long
Dclk1-
Short
q-PCR Dclk1-Long
ladder
FNS cells
Oli-neu cells
2500 bp
2000 bp
q-PCR Dclk1-Short
FNS cells
Oli-neu cells
ladder
400 bp
300 bp

Figure 5

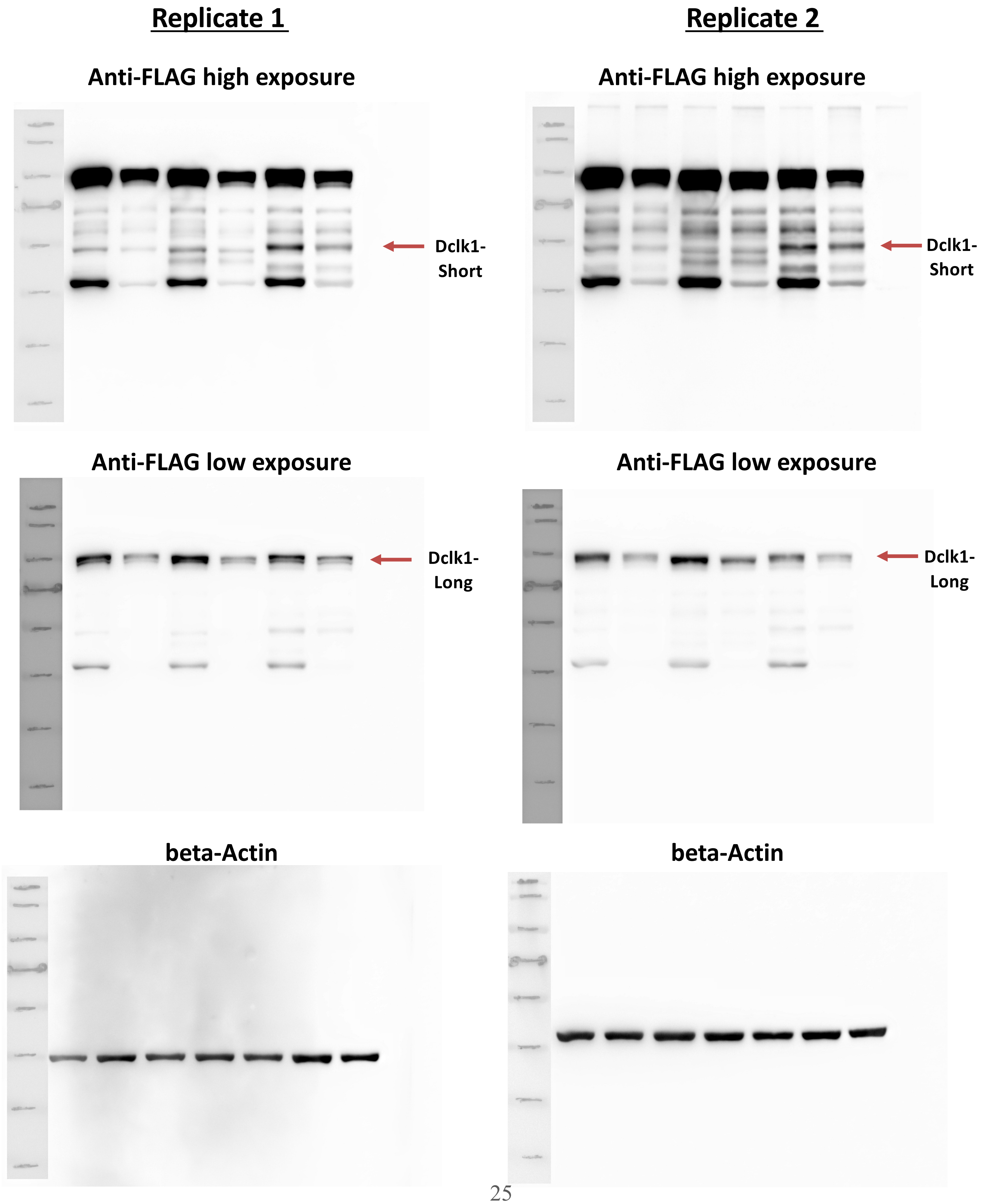

Replicate 1
Replicate 2
Anti-FLAG high exposure
Anti-FLAG high exposure
Dclk1-Short
Dclk1-Short
Anti-FLAG low exposure
Anti-FLAG low exposure
Dclk1-Long
Dclk1-Long
beta-Actin
beta-Actin

Figure 5

Replicate 3

Anti-FLAG high exposure

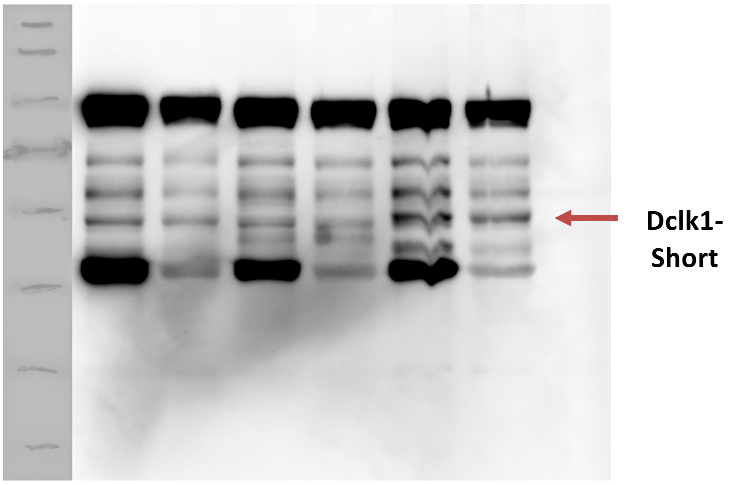

Anti-FLAG low exposure

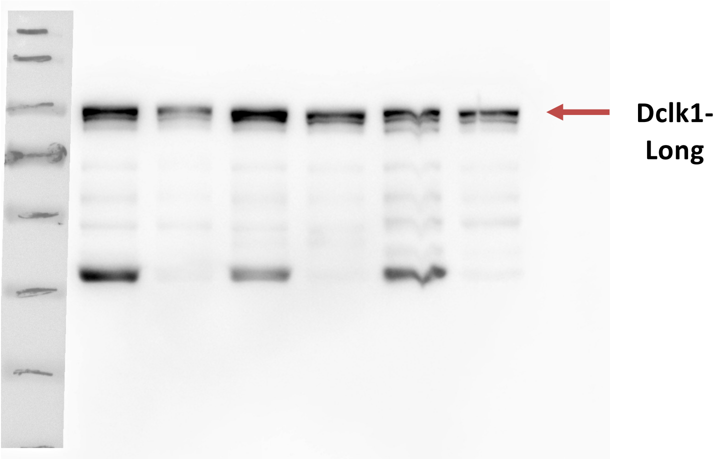

beta-Actin

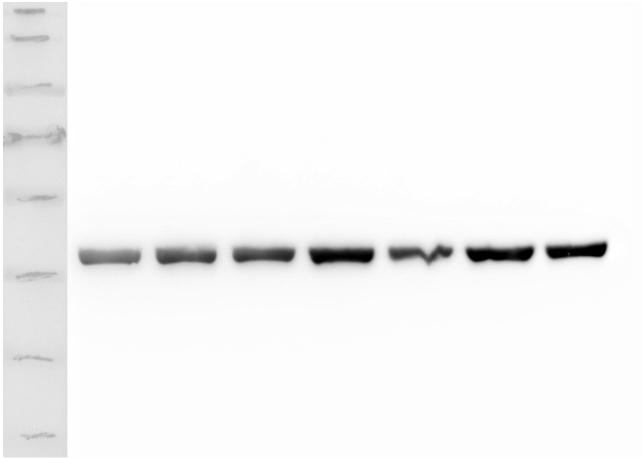

Figure 6

Anti-MYC

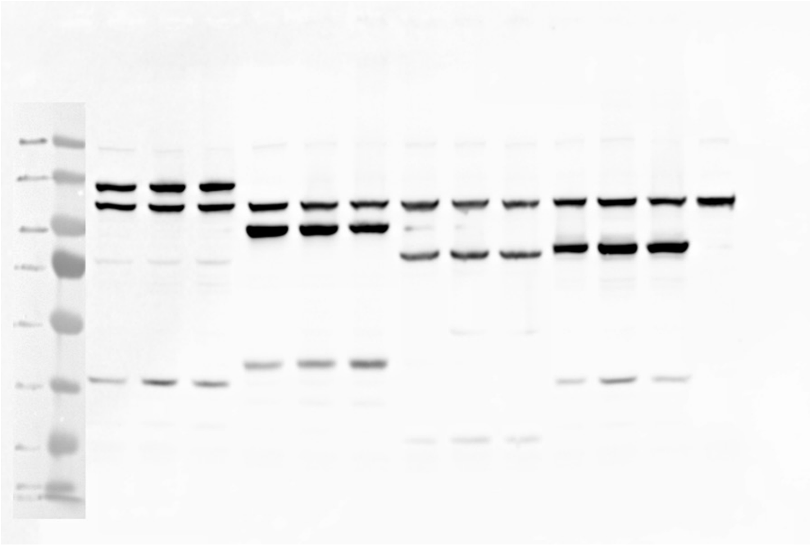

Streptavidin-HRP

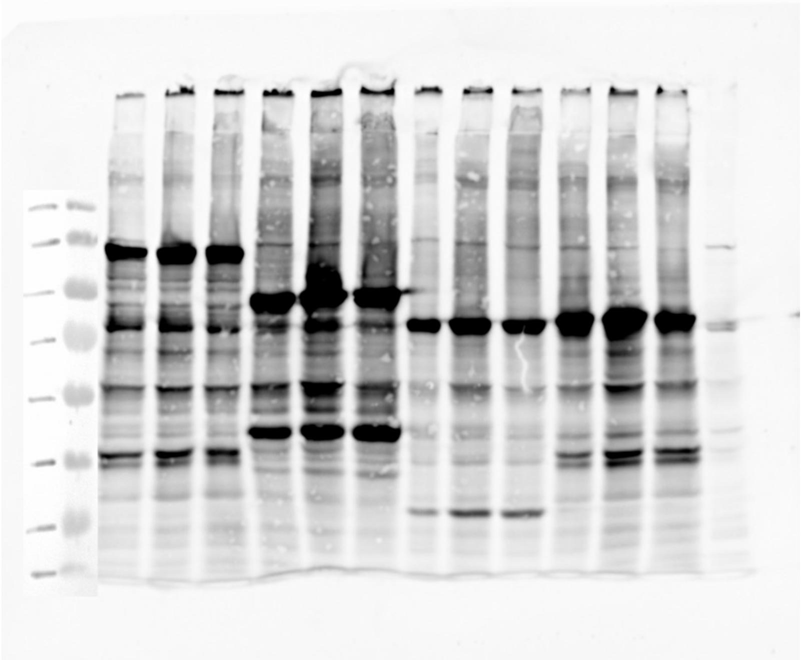

Figure S7

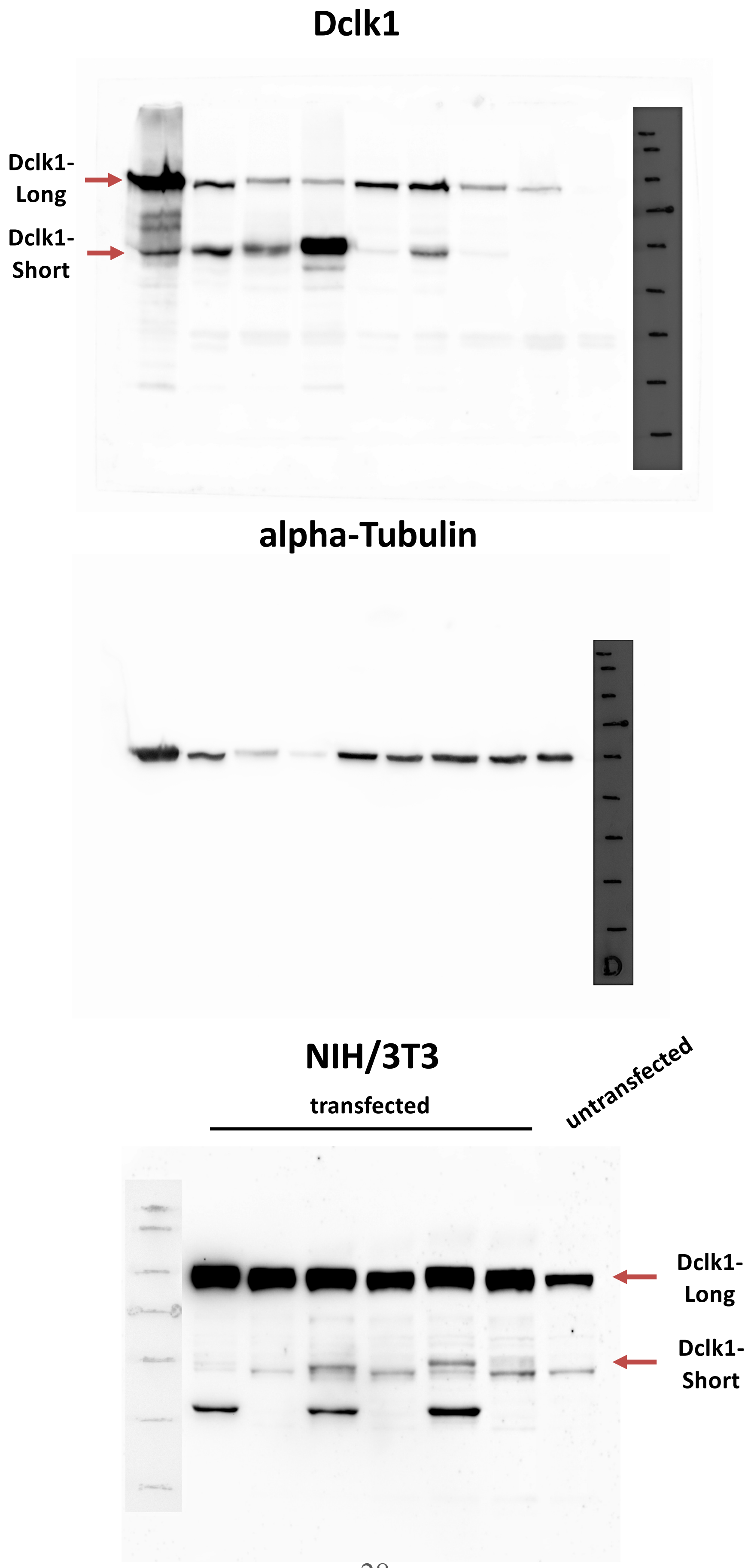

Dclk1
Dclk1-Long
Dclk1-Short
alpha-Tubulin
NIH/3T3
transfected
untransfected
Dclk1-Long
Dclk1-Short
